# Splicing factor FUS facilitates the progression of PIT1-lineage PitNETs by upregulating MDM2

**DOI:** 10.7150/thno.124068

**Published:** 2026-01-01

**Authors:** Xu Wang, Jiang Li, Chenggang Jiang, Chengkai Zhang, Linhao Yuan, Tieqiang Zhang, Yuqi Liu, Shunchang Ma, Peng Kang, Deling Li, Xiudong Guan, Jian Chen, Wang Jia

**Affiliations:** 1 Department of Neurosurgery, Beijing Tiantan Hospital, Capital Medical University, Beijing, 100070, China.; 2 China National Clinical Research Center for Neurological Diseases, Fengtai, Beijing, 100070, China.; 3 Beijing Neurosurgical Institute, Beijing, 100070, China.; 4 Chinese Institute for Brain Research, Beijing, Research Unit of Medical Neurobiology, Chinese Academy of Medical Sciences, Beijing, 102206, China.

**Keywords:** FUS, PitNETs, splicing, MDM2, ASO

## Abstract

**Background:** Splicing factors play pivotal roles in mRNA processing and are implicated in tumor progression. The aberrant expression of splicing factors is closely associated with the invasiveness and secretion profiles of pituitary neuroendocrine tumors (PitNETs). In this study, we explored the involvement of splicing factors in PIT1-lineage PitNET progression and assessed the feasibility of targeting the splicing process as a therapeutic approach.

**Methods:** Statistical data on PitNET subtypes were obtained from the National Brain Tumor Registry of China (NBTRC), and gene expression analysis was conducted on 40 clinical samples collected for this study. Transcriptome analysis and RNA immunoprecipitation sequencing (RIP-seq) were utilized to examine FUS-mediated alternative splicing and to identify mRNA binding sites in PitNET cells. Minigene splicing assays were employed to confirm the specific exonic and intronic regions. Additionally, Annexin V/PI assays and JC-1 staining were conducted to evaluate apoptosis.

**Results:** The expression of the splicing factor FUS was elevated in PIT1-lineage PitNETs and was correlated with increased proliferative capacity and reduced apoptosis levels. Transcriptome sequencing revealed that the knockdown of FUS led to extensive exon skipping and activated the p53 pathway. In addition to RIP-seq analysis, these findings suggest that FUS contributes to the inclusion of exon 3 to generate full-length MDM2, a well-established negative regulator of p53. Antisense oligonucleotides (ASOs) specifically designed to target binding sequences on pre-mRNAs effectively disrupted the FUS-mediated splicing process, consequently impeding the progression of PitNETs.

**Conclusions:** Our study elucidated the critical function of FUS as a splicing factor in PitNETs. Furthermore, we illustrated that targeting the splicing mechanism associated with MDM2 could restore p53 levels, thereby impeding the progression of PitNETs. This discovery presents a potentially novel strategy for the clinical management of PIT1-lineage PitNETs.

## Introduction

Pituitary neuroendocrine tumors (PitNETs) constitute the second most prevalent category of central nervous system tumors, accounting for 15% of all primary brain tumors. Clinically, PitNETs have an annual incidence of approximately 40 cases per million individuals 1. Approximately 48% of PitNETs are macroadenomas (>10 mm) and can exert a mass effect, potentially leading to visual-field deficits, headaches or hypopituitarism 2. PitNETs include both functioning tumors, which are distinguished by their secretion of pituitary hormones, and nonfunctioning tumors.

The classification of PitNETs involves identifying the cell type through the characterization of hormone genes and cell lineage-specific transcription factors. This classification encompasses the PIT-1 (POU1F1) lineage, which includes somatotroph, lactotroph, and thyrotroph cells; the T-PIT (TBX19) lineage, which pertains to corticotroph cells; the SF-1 (NR5A1) lineage, which is associated with gonadotroph cells; as well as null-cell and plurihormonal tumors 3,4. Single-cell multiomics profiling has revealed the inherent heterogeneity among PitNET subtypes, distinct immune microenvironments and varying recurrence rates 5-7. PIT1-lineage tumors represent approximately 40% of all PitNETs 8. Pharmacological therapies include dopamine D2-receptor agonists for PRL PitNETs and somatostatin analogs or growth hormone receptor antagonists for GH PitNETs; however, 30-70% of patients eventually require surgical intervention due to pharmacological resistance 9. Excessive tumor size or close proximity to critical structures, especially the internal carotid artery and optic nerve, serves as an independent predictor of postoperative recurrence 10. Despite recent advancements 11, the primary clinical challenges in managing PIT1-lineage tumors remain the control of tumor volume and the achievement of sustained endocrine remission.

Alternative splicing (AS) of precursor mRNA (pre-mRNA) represents a crucial mechanism for enhancing the diversity of gene expression. RNA sequencing analyses have revealed that more than 90% of human protein-coding genes are subject to pre-mRNA splicing 12. Splicing factors play crucial roles in the regulation of alternative splicing by interacting with cis-elements in pre-mRNAs to either promote or suppress the splicing process 13. Significant heterogeneity and dysregulation in splicing have been observed across all subtypes of PitNETs 14. Mutations in the splicing factor SF3B1 result in aberrant splicing, which not only disrupts tumorigenesis and hormone secretion but also increases invasive potential 15,16. With increasing insight into splice factor dysregulation in tumors 17, splice-targeting therapeutics are being actively developed. Antisense oligonucleotides (ASOs) are short, single-stranded nucleic acids that bind to specific mRNA targets to modulate gene expression. ASOs designed to modulate pre-mRNA splicing are currently FDA approved for the treatment of spinal muscular atrophy 18. Furthermore, the use of ASOs has emerged as a promising strategy for the treatment of gliomas and diffuse intrinsic pontine gliomas 19,20. However, our understanding of the aberrant regulation of splicing factors within PIT1-lineage PitNETs remains limited, and novel therapeutic strategies are urgently needed.

FUS is classified as a nucleic acid-binding protein that is primarily localized within the nucleus. 21. FUS dysfunction has been conclusively associated with a range of neurodegenerative disorders, most notably amyotrophic lateral sclerosis, and is currently acknowledged as a critical therapeutic target 22. FUS orchestrates neuronal plasticity through the modulation of RNA splicing and trafficking 23, and its dysregulated activation contributes to the progression of glioma 24. Here, we report that FUS is overexpressed in PIT1-lineage PitNETs compared with normal pituitary tissue. Furthermore, PitNETs with elevated FUS expression are associated with a significantly increased risk of postoperative hypopituitarism. We additionally demonstrated that FUS facilitates the proliferation of PIT1-lineage PitNETs and the growth of xenograft tumors. Mechanistically, we report that FUS promotes the inclusion of exon 3 in *MDM2* pre-mRNA, thereby increasing MDM2 levels and suppressing p53 activity. These findings identify FUS as an essential oncogenic splicing factor with therapeutic potential in PIT1-lineage PitNETs.

## Materials and Methods

### Ethics statement

The conduct of this study was fully aligned with prevailing guidelines. The National Brain Tumour Registry of China (NBTRC) was approved by the ethics committee of Beijing Tiantan Hospital (KY 201912402). Human PitNET specimens were procured from surgical procedures conducted on patients at Tiantan Hospital. The Ethics Committee of Tiantan Hospital approved the study protocols (KY202215501). Normal pituitary gland specimens were obtained from Human Brain Bank of Peking Union Medical College. Written informed consent was obtained from all participating patients. The animal studies were approved by the Animal Ethics Committee (BNI202304005).

### Immunohistochemistry (IHC)

Primary antibody incubation was performed on 5 μm tissue sections obtained from paraffin-embedded samples. For signal detection, horseradish peroxidase (HRP)-conjugated secondary antibodies with DAB substrate were used. The primary antibodies used included anti-FUS (1:200 dilution, 11570-1-AP; Proteintech), anti-Ki67 (1:100 dilution, 28074-1-AP, Proteintech), anti-MDM2 (1:100 dilution, 2524S, Cell Signaling Technology), anti-P53 (1:100 dilution, 51541S, Cell Signaling Technology), and anti-BAX (1:100 dilution, 50599-2-Ig, Proteintech). Histological scoring was performed through independent evaluations of the positive cell proportion and staining intensity.

### Cell culture

The primary rat GH3 and MMQ cell lines were purchased from Procell (Wuhan, China) and cultured in Ham's F-12K (Thermo Fisher Scientific) supplemented with 10% fetal bovine serum and 10% horse serum (Thermo Fisher Scientific). Patient-derived primary PitNETs were cultured in DMEM/F-12 medium (Gibco) supplemented with 2% B-27 (Thermo Fisher Scientific), 1% N-2 (Thermo Fisher Scientific), 20 ng/mL epidermal growth factor (EGF; PeproTech), 10 ng/mL basic fibroblast growth factor (bFGF; PeproTech), 0.5% GlutaMAX (Thermo Fisher Scientific), 2% HEPES, 20 ng/mL glucose and 2-mercaptoethanol (β-ME; Thermo Fisher Scientific).

### Vector construction and transfection

Cells were transiently transfected using Lipofectamine 2000 (Thermo Fisher Scientific) according to the manufacturer's protocol. For siRNA knockdown, cells were transfected with 100 pmol of siRNA (GenePharma) for 48 h. The FUS-shRNA, FUS-overexpressing and negative control lentivirus were purchased from Obio Technology. The siRNAs sequences for FUS and MDM2 in rat, were the following: siFUS-1: 5'-GCCAAGAUCAGUCCUCUAU-3', siFUS-2: 5'-CGUGGUGGCUUCAAUAAAU-3', siMDM2-1: 5'-CGUGCAAGCAUCACAAGAA-3', siMDM2-2: 5'-CCUCGUGCAAUGAAAUGAA-3'. The sequence of siRNA for human FUS was as follows: 5'-CGGACAUGGCCUCAAACGA-3'. Expression constructs for shNC, shFUS, NC, MDM2, MDM2 ΔExon3, FLAG-FUSwt (FUS), FLAG-FUS mutation (FUSmut1), FLAG-FUS mutation (FUSmut2) were purchased from Obio Technology. The MOE-modified ASO sequences were synthesized by Beijing DIA-UP Biotechnology Co, Ltd. The remaining sequences are listed in Table S1.

### RNA isolation and PCR analysis

Total RNA was isolated and DNase-treated (ES Science) before reverse transcription into cDNA using a Toyobo qPCR RT Kit. Quantitative PCR was performed on the resulting cDNA using Taq Pro Universal SYBR qPCR Master Mix (Vazyme). The mRNA levels were normalized to those of glyceraldehyde-3-phosphate dehydrogenase (*GAPDH*). Semiquantitative RT‒PCR was performed to analyze alternatively spliced isoforms. Primers were designed for the targeted exons, and 2× Taq Master Mix (P222-03-AA, Vazyme) was used to amplify target exon spliced-in or spliced-out isoforms. Multiplex probe-based PCR was used to quantify splice isoform expression levels, utilizing specific probes for isoforms and Probe Master Mix (QN114-01, Vazyme) as the reaction buffer. The primer sequences are provided in Table S1.

### Cell proliferation assays

Cell viability was evaluated in a CCK-8 assay (Beyotime) in accordance with the manufacturer's protocol. Cells were plated in 96-well plates and incubated with CCK-8 reagent at 37 °C for 1 hour, after which the absorbance was measured at 450 nm. The initiation of transfection served as the 0-hour point. The influence of the siRNAs on cellular proliferation was systematically assessed over a duration of four days, and the half-maximal inhibitory concentration (IC50) of the ASOs was measured 48 h after treatment. EdU cell proliferation assays were conducted using the Cell-Light EdU Apollo567 *In Vitro* Kit (RiboBio) following the protocols provided by the manufacturer.

### Flow cytometry

The cell cycle distribution was analyzed by ethanol fixation (75%, 4 °C, 48 h) followed by propidium iodide (PI; BD Biosciences) staining for 15 min. Apoptosis was assessed via Annexin V-FITC/PI (BD) double staining after the cells were washed with PBS. The mitochondrial membrane potential was measured using a JC-1 kit (Beyotime). All the samples were processed on a Fortessa cytometer (BD) and analyzed with FlowJo (v10).

### Immunoprecipitation (IP) and western blotting (WB)

Total protein lysates were obtained in protease inhibitor-supplemented IP lysis buffer (Thermo Fisher Scientific, Sigma‒Aldrich). For immunoprecipitation, the lysates were incubated overnight at 4 °C with anti-ubiquitin (1:100, 3936S, Cell Signaling Technology), anti-FLAG (1:100, 8146S, Cell Signaling Technology), or control IgG (1:100, ab172730, Abcam), followed by a 2-hour incubation at room temperature with Protein A/G magnetic beads (Thermo Fisher Scientific). Eluted proteins were subsequently analyzed by western blot or mass spectrometry. For immunoblotting, the samples were resolved via 10% SDS‒PAGE (PG112, Epizyme), transferred to PVDF membranes, blocked with 5% skim milk, and probed overnight at 4 °C with primary antibodies. Detection was performed using HRP-conjugated secondary antibodies with an enhanced chemiluminescence (ECL) system. The following primary antibodies were used: anti-FUS (1:1000, 11570-1-AP, Proteintech), anti-MDM2 (1:1000, 2524S, Cell Signaling Technology), anti-P53 (1:1000, 51541S, Cell Signaling Technology), anti-BAX (1:1000, 50599-2-Ig, Proteintech), anti-GAPDH (1:5000, 10494-1-AP, Proteintech), and anti-FLAG (1:1000, 8146S, Cell Signaling Technology).

### Immunofluorescence and FISH

After being cultured on glass coverslips and fixed with 4% paraformaldehyde, the cells were immunostained for FUS and SC-35 to explore their subcellular distribution with the following antibodies: anti-Phalloidin (1:100 dilution, ab176757, Abcam), anti-FUS (1:100 dilution, 11570-1-AP, Proteintech) and anti-SC-35 (1:100 dilution, ab11826, Abcam). To detect MDM2 EXON 3, a CY3-modified fluorescence *in situ* hybridization (FISH) probe mixture was used in accordance with the manufacturer's protocol (GenePharma).

### RNA sequencing and bioinformatics analysis

RNA sequencing was conducted on human PitNET samples (n = 115) and their corresponding normal pituitary controls (n = 10) by LC Bio Corporation (Hangzhou, China). The Wilcoxon test was used to identify differentially expressed genes, with genes whose log2FC was > 0.5 and whose *P* value was < 0.05 selected for further analysis. These selected genes were subsequently filtered according to the baseline expression levels of splicing factors. Illumina PE150 sequencing was employed for transcriptome analysis of the GH3 and MMQ lines, with each group comprising three independent biological replicates sequenced in paired-end mode. Differential gene expression was identified using cutoffs of |log2(FC)| > 1 and *P* < 0.05.

Heatmaps were generated utilizing the Hiplot platform. Differential expression and pathway analyses were conducted through gene set enrichment analysis (GSEA). Alternative splicing (AS) events were identified using rMATS (version 4.1.2). Following the classification of AS events into five types, significance cutoffs (p < 0.05, |IncLevelDifference| > 0.1) were applied. Enrichment of FUS-binding signals flanking regulated exons was subsequently quantified using DeepTools (v3.5.5). Gene Ontology (GO) enrichment analysis and Kyoto Encyclopedia of Genes and Genomes (KEGG) analysis were conducted, and the results were visualized using R software (version 4.4.3). The sequencing data were examined using the Integrative Genomics Viewer (IGV) to facilitate the visualization of exon skipping events. Sequencing reads were aligned to the Rn6 reference genome with HISAT2 (version 2.2.0), followed by sorting using SAMtools (version 1.9).

### RNA immunoprecipitation (RIP) assay

GH3 and MMQ cells were lysed and subjected to RIP with an EZ-Magna kit (17-701, Merck Millipore). After overnight incubation at 4 °C with magnetic beads coated with anti-FUS antibody (11570-1-AP, Proteintech), the immunocomplexes were washed 6 times and digested with proteinase K. Total RNA was isolated with phenol/chloroform/isoamyl alcohol (125:24:1), followed by qPCR or sequencing (LC Bio Corporation). Input was used for normalization.

### Minigene assay

The fragment spanning introns 2-3 of the FUS gene was cloned and inserted into the pSPL3 vector. The recombinant constructs, designated pSPL3-FUS-WT and pSPL3-FUS-MUT, were transiently transfected into the GH3 and MMQ cell lines with X-tremeGEN HP transfection reagent (Roche). After 48 h, RNA was extracted and subsequently reverse-transcribed into cDNA. Semiquantitative RT‒PCR was employed to analyze alternatively spliced isoforms as previously described.

### Nude mouse xenograft model

Male BALB/c nude mice (4 weeks old) were purchased from Beijing Weitong Lihua Experimental Animal Technical Co., Ltd., and maintained under pathogen-free conditions at 24 °C with a 12-hour light‒dark cycle. The mice were subcutaneously injected with GH3 and MMQ cells. Anesthesia was administered at the study endpoint, after which the tumor volume and weight were recorded. Intratumoral injection of ASO was administered two weeks after subcutaneous injection. The mice were randomly divided into two groups (n = 8/group) and treated every 72 hours with ASO-Lipo2000 complexes (5 nmol ASO in 3 μl of Lipo2000 with 25 μl of Opti-MEM). Subcutaneous tumor samples were fixed in formalin following harvest and then processed for HE staining and immunohistochemistry.

### Statistical analysis

The CGGA-CNS pituitary tumor database is available at https://cgga-cns.org.cn. Pearson correlation analysis was used to evaluate the relationships between gene expression levels, and progression-free survival (PFS) curves were generated and compared using the log-rank test. Statistical significance was evaluated using the chi-square test, Student's t test, and one- and two-way ANOVA, which were conducted with GraphPad Prism 9.0 software. Data for each group are presented as the mean ± standard error of the mean (SEM), and a p value of less than 0.05 was considered to indicate statistical significance.

## Results

### Elevated FUS suggests a poor postoperative prognosis in patients with PIT1-lineage PitNETs

**Figure 1:**
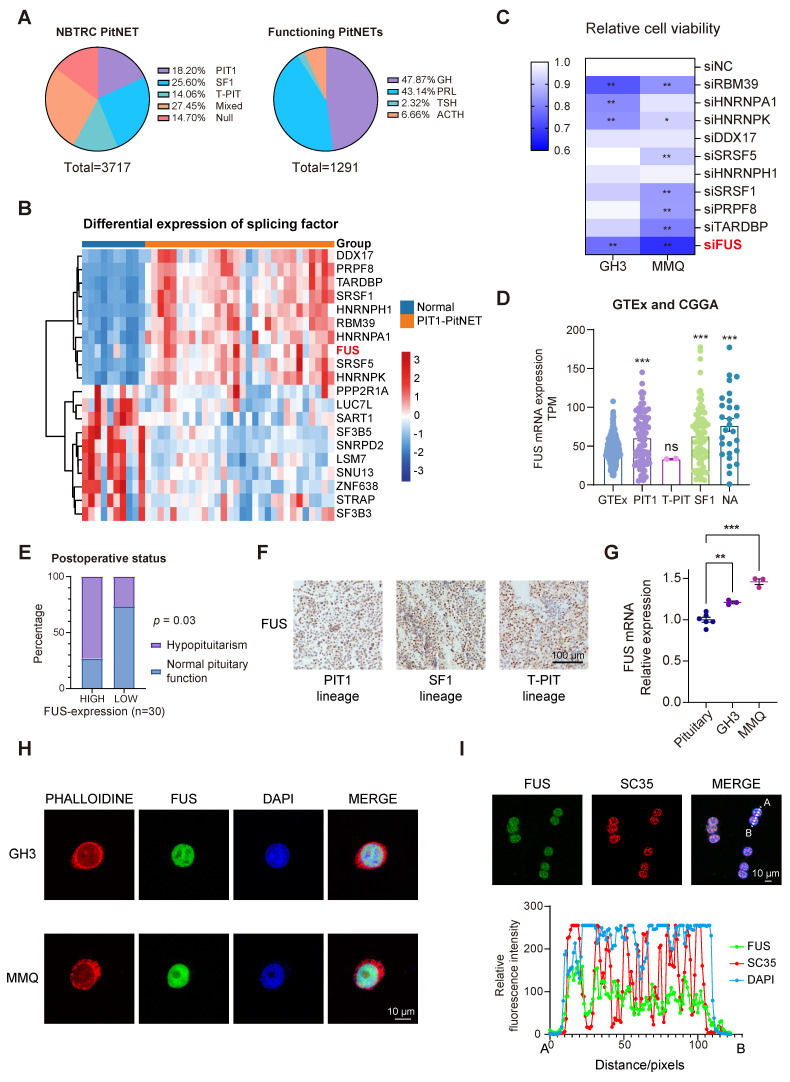
** PIT1-lineage PitNETs exhibit increased expression of the splicing factor FUS. A** The pie charts showing the proportions of transcription-factor lineage of PitNETs (n=3717), and the distribution of hormonal subtypes specifically in functional PitNETs (n=1291). Data were obtained from the NBTRC cohort. **B** Differential expression profiles of splicing-related genes in PIT1-lineage PitNETs (n=30) versus normal pituitary specimens (n=10). **C** Cell-viability assessment by CCK-8 assay following individual knockdown of splicing factors (n=3). **D** Analysis of FUS expression using GTEx datasets (n=283) and CGGA-CNS Pituitary Databases (n=190). The NA group comprised samples annotated as "NA" and "silent" in the database. **E** Chi-square analysis of the relationship between FUS expression levels and the postoperative hypopituitarism (n = 30). **F** IHC staining of FUS in different lineage of PitNETs (n = 8). **G** PCR analysis of FUS mRNA expression in Rat pituitary gland and 2 PIT1-lineage cell lines (n = 3). **H** Immunofluorescence reveals FUS (green) subcellular distribution in GH3 and MMQ cells. Nuclei (blue, DAPI) and F-actin (red, phalloidin) were visualized (n=3). Scale bar = 10 μm. **I** Immunofluorescence images for FUS (green) and SC35 (red) staining for GH3 cells (n = 3). Scale bar = 10 μm. Data are shown as mean ± SEM. *P < 0.05, **P < 0.01, ***P < 0.001.

The NBTRC is the first multihospital-based brain tumor registry in China 25. To quantify the clinical surgical burden associated with various PitNET subtypes, we analyzed 3,717 consecutively enrolled patients from Beijing Tiantan Hospital and the NBTRC from 2011-2021, all of whom had undergone definitive histopathological staining. Although PIT1-lineage tumors are the second most prevalent type among all PitNETs (Figure S1A-B), they constitute the majority when functional adenomas are considered (Figure 1A). Given its relatively high postoperative recurrence rate 26, novel strategies for PIT1-lineage PitNETs need to be implemented. A total of 115 PitNET samples and 10 normal samples were sequenced, with subsequent focused analysis of the 30 PIT1-lineage PitNETs (Table S2). To explore the potential of splicing factors in PIT1-lineage PitNETs, we compared the expression levels of 362 mRNA-splicing proteins 27 with those in normal pituitary tissue (Figure 1B and Figure S1C-D). Among the significantly upregulated splicing factors, we focused on the ten most abundant proteins for RNAi screening (Figure S1E). In the rat PIT1-lineage adenoma cell lines GH3 (somatotroph subtype) and MMQ (lactotroph subtype), the downregulation of FUS resulted in a significant reduction in cell viability (Figure 1C and Figure S1F).

The relationships between FUS expression and clinical features were further investigated. Integrative analysis of the CGGA-CNS PitNETs database and GTEx normal pituitary samples also revealed elevated FUS expression across various PitNET lineages (Figure 1D). In patients with PIT1-lineage PitNETs, those whose tumors exhibited elevated levels of FUS demonstrated a significantly increased incidence of postoperative hypopituitarism (Figure 1E and Table S3). Although the average tumor volume was greater in the high-FUS-expression group than in the low-FUS-expression group, there was no significant difference in tumor volume or postoperative PFS in the limited sample size group (Figure S1G-H). In the cohort of 115 samples across all lineages, patients with elevated FUS expression were significantly older and exhibited larger tumor volumes and increased incidence rates of visual impairment, suprasellar invasion, and postoperative hypopituitarism (Table S4). To avoid intrinsic differences among multiple lineages, we conducted our subsequent investigation exclusively on the PIT1 lineage. Although no significant upregulation in FUS levels was observed in the PIT1 lineage compared with the other lineages (Figure 1F, Figure S1I-J and Table S5). Compared with normal rat pituitary, the PIT1-lineage GH3 and MMQ cell lines demonstrated significantly increased FUS levels (Figure 1G).

FUS was observed to be exclusively localized within the nucleus (Figure 1H) and simultaneously exhibited significant colocalization with the splicing-associated marker SC35 (Figure 1I) 28. Collectively, these findings demonstrate that high FUS expression predicts poor postoperative outcomes in patients with PIT1-lineage PitNETs. These findings underscore the pressing demand for the development of novel therapeutic strategies.

### Knockdown of FUS inhibits growth and hormone secretion in PIT1-lineage PitNETs

**Figure 2:**
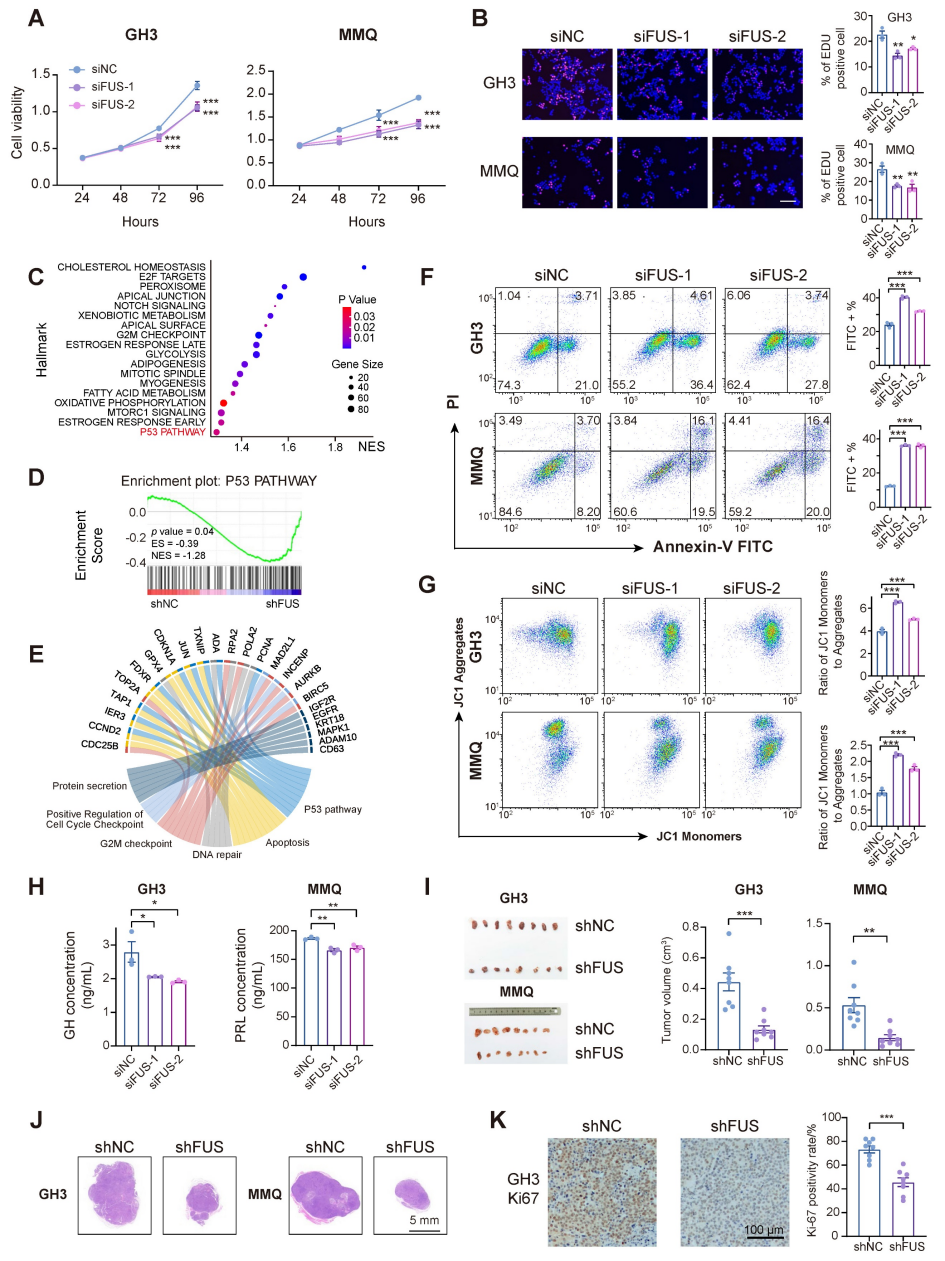
** In PIT1-lineage cells, FUS knockdown attenuates proliferative and secretory capacity and activates p53 signaling and apoptotic pathways. A** CCK-8 assay evaluated proliferation of GH3 and MMQ cells treated with control siNC or 2 distinct siRNA (n = 3). **B** Cell proliferation was quantified by EdU assay in GH3 and MMQ cells following transfection with control siNC or 2 distinct siRNA (n = 3). Scale bar = 100 μm. **C** GSEA analysis were conducted based on the transcriptome from GH3 cells after FUS knockdown. **D** p53 pathway in GSEA analysis from GH3 cells after FUS knockdown. **E** Circos plot analysis of related pathway in GSEA analysis. **F** Apoptosis was quantified by Annexin V-FITC/PI flow cytometry in GH3 and MMQ cells transfected with control siNC or 2 distinct siRNA (n = 3). **G** JC-1 labeling and flow cytometry assessed mitochondrial membrane potential in GH3 and MMQ cells transfected with control siNC or 2 distinct siRNA (n = 3). **H** ELISA assay for GH and PRL levels in the culture supernatants of GH3 and MMQ cells respectively, and cell numbers were normalized across different groups (n = 3). **I** Representative tumor image and tumor volume of subcutaneous xenograft experiments of GH3 and MMQ cell lines with FUS knockdown (n = 8 mice per group). **J** Representative images of HE staining of subcutaneous xenografts of GH3- or MMQ-shNC or -shFUS. Scale bar = 5 mm. **K** Representative Ki67 immunostaining and quantitative analysis of subcutaneous xenografts derived from GH3-shNC and GH3-shFUS cells. Scale bar = 100 μm. Data are shown as mean ± SEM. *P < 0.05, **P < 0.01, ***P < 0.001.

Two independent siRNAs were used to knock down FUS in different PIT1-lineage cell lines to investigate its potential role in PIT1-lineage PitNETs (Figure S2A-B). Following transfection with FUS-targeting siRNAs, the cell viabilities of the GH3 and MMQ cell lines significantly decreased (Figure 2A). The inhibition of FUS resulted in decreased proliferation of the GH3 and MMQ cells (Figure 2B).

We subsequently generated stable GH3 and MMQ cell lines expressing shNC or shFUS via lentiviral infection and performed transcriptome analyses by RNA sequencing (Figure S2C). GSEA of the hallmark pathways revealed significant alterations following FUS knockdown (Figure 2C). Notably, the activity of the classical tumor-suppressor p53 pathway substantially increased in shFUS cells (Figure 2D and Figure S2D). In PitNETs, abnormal expression of p53 is correlated with an unfavorable prognosis and is involved in modulating cellular proliferation and apoptotic processes 29,30. The differentially expressed genes identified through RNA-seq were associated with p53-related pathways, such as apoptosis, DNA repair, and cell cycle regulation, as well as pathways involved in pituitary hormone secretion (Figure 2E). Informed by the enriched pathways and the well-established role of p53 in tumorigenesis 31, we concentrated on assessing the effects of FUS knockdown on cell cycle progression and apoptosis. Flow cytometric analysis of cell cycle parameters revealed reductions in the proportions of GH3- and MMQ-shFUS cells in the S phase, with the magnitudes of change remaining within 5% (Figure S2E). Moreover, apoptosis assays revealed significant increases in both GH3 and MMQ cell staining with Annexin-V and PI following FUS knockdown (Figure 2F). Flow cytometric subgating analysis revealed an approximately 10% increase in early apoptotic cells. This observation was further validated by increased staining for JC-1, a mitochondrial marker that is indicative of early apoptosis (Figure 2G). These findings collectively indicate that FUS may influence PitNETs via a p53-dependent apoptotic pathway.

To examine the effects of FUS on hormone secretion in PIT1-lineage cells, hormone concentrations in the supernatant medium were quantified by enzyme-linked immunosorbent assay (ELISA). The results demonstrated that FUS knockdown led to significant reductions in the secretion levels of growth hormone (GH) and prolactin (PRL) in the GH3 and MMQ cell lines, respectively (Figure 2H). Moreover, the expression of PIT1, a key transcriptional regulator of hormone secretion, was markedly reduced following the knockdown of FUS, which was further corroborated by GSEA and Gene Ontology (GO) analysis (Figure S2F-H).

To assess the *in vivo* effects of FUS deficiency on PIT1-lineage PitNETs, GH3- and MMQ-shFUS and shNC cells were subcutaneously injected to establish xenograft models (n = 8/group). The downregulation of FUS in the GH3 and MMQ cell lines resulted in a significant reduction in tumor volume (Figure 2I-J). IHC analysis indicated that compared with the control treatment, the suppression of FUS expression significantly reduced the Ki-67 proliferation index (Figure 2K and Figure S2I-J).

### Genome-wide landscape of FUS-regulated splicing events in PIT1-lineage PitNETs

**Figure 3:**
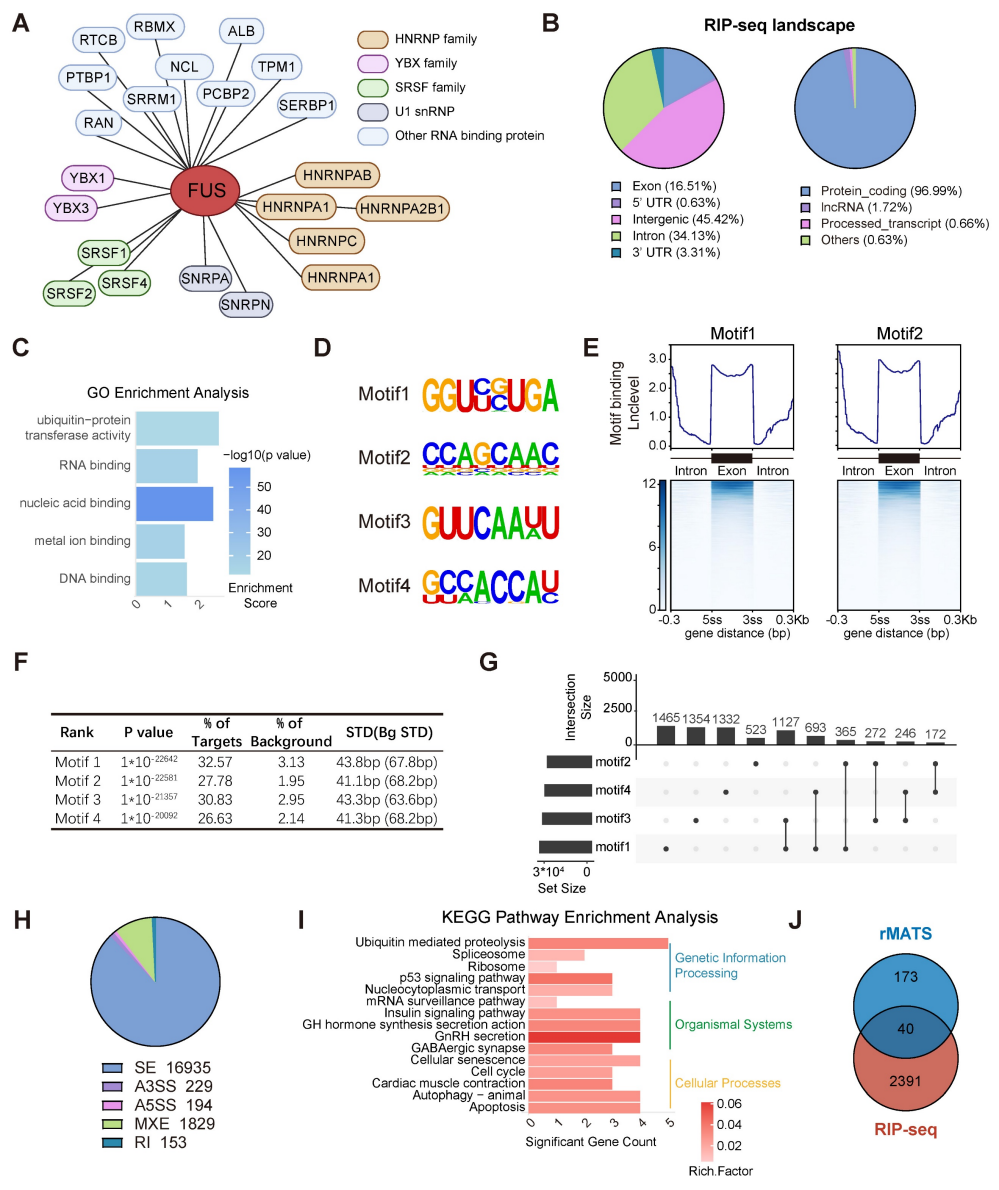
** Genome-wide landscape of FUS-regulated splicing events in PIT1-lineage PitNETs. A** Biological analysis of FUS-interacting proteins in GH3 and MMQ cells using immunoprecipitation followed by mass spectrometry. **B** Analysis of the distribution of FUS-binding reads in different genomic regions and biological categories of binding substrates by RIP-seq. **C** Gene Ontology analysis of genes bound by FUS in RIP-seq. **D** Enriched sequential elements of the top-four FUS-binding motifs. **E** Heat map showing FUS-binding motif distribution in the exon region and 300 bp around the 3' or 5' splice site junction, generated using DeepTools. Exon lengths were normalized to 300 bp. **F** De novo motif analysis of FUS and the top-4 motifs ranked according to the p-values calculated by HOMER. **G** Upset plot illustrating the distribution of motifs 1-4 within the FUS-binding regions. Set size indicates the number of genes with specific motif, and intersection size reflects the number of genes with a particular combination of motifs. **H** Pie chart showing the distribution of various AS types in transcriptome data derived from GH3 cells following FUS knockdown. **I** KEGG enrichment analysis of genes exhibiting significant differential AS following FUS knockdown. **J** Venn diagram of 2391 FUS-binding genes from RIP-seq with 173 genes exhibiting significant AS events in both GH3 and MMQ cell lines. The intersection size represents the number of genes both bound by FUS and regulated by alternative splicing.

To elucidate the role of FUS in the splicing process, we initially identified proteins associated with FUS through immunoprecipitation coupled with mass spectrometry in the GH3 and MMQ cell lines (Figure S3A). We identified FUS in association with the SRSF family and U1 snRNP proteins, which are canonical components of the spliceosome, thereby indicating its involvement in the core splicing machinery (Figure 3A and Figure S3B).

RIP-seq analysis of FUS was conducted in GH3 cells to obtain comprehensive insights into FUS-RNA binding sites. The analysis revealed that more than 96% of FUS-binding targets were associated with protein-coding genes, with FUS occupancy observed in both intronic and exonic regions of pre-mRNAs (Figure 3B and Figure S3C). GO analysis of FUS target genes for molecular function revealed significant enrichment of nucleic acid-binding proteins and ubiquitin-related activities (Figure 3C). To enhance the characterization of FUS interactions with RNA, we employed the HOMER algorithm to identify RNA motifs recognized by FUS (Figure 3D). We conducted an analysis of FUS binding intensity across both exonic and intronic regions and found that FUS predominantly binds to entire exonic regions, as well as to intronic regions that are situated away from splice junctions (Figure 3E-F and Figure S3D). Furthermore, compared with individual motifs, FUS-binding motifs were more frequently observed as combinations (Figure 3G).

rMATS analysis was employed to investigate FUS-controlled AS events using RNA-seq data derived from GH3 and MMQ cells with FUS knockdown. Five primary events of alternative splicing have been identified: exon skipping, mutually exclusive exons, alternative 5′ splice site usage, alternative 3′ splice site usage, and intron retention. Notably, exon skipping constituted 87% of all the observed splicing events (Figure 3H and Figure S3E). KEGG enrichment analysis of significant splicing alterations revealed that FUS-mediated splicing dysregulation substantially disrupted the p53 signaling pathway and hormone secretion processes, corroborating our previous observations (Figure 3I). To identify the direct targets associated with FUS function in PitNETs, we analyzed the subset of FUS-bound genes in RIPseq that concurrently exhibited significant splicing alterations (Figure 3J and Table S6). Collectively, these findings elucidate the splicing pattern and downstream candidates associated with the splicing factor FUS in PitNETs.

### FUS facilitates the inclusion of exon 3 in *MDM2* pre-mRNA by direct binding

**Figure 4:**
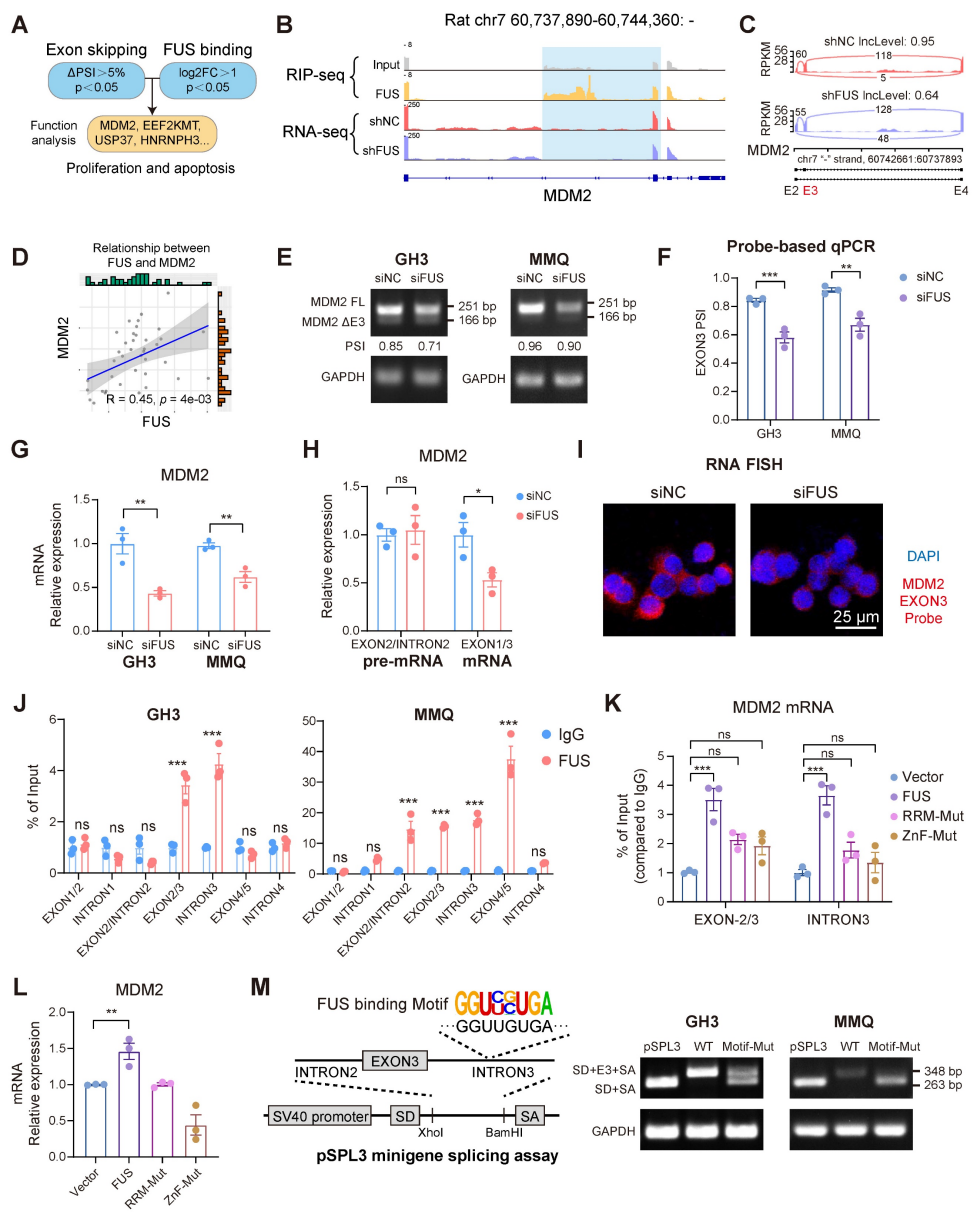
** FUS facilitates the inclusion of exon 3 in MDM2 pre-mRNA by directly binding. A** Systematic strategy for the identification of FUS-regulated substrates. **B** Visualization the AS events of MDM2 and its FUS-binding region, integrating RNA-seq and RIP-seq tracks with IGV; the blue shading highlights both the AS event and the FUS-bound segment. **C** IGV-Sashimi plots illustrating the AS events in MDM2 using transcriptome data in GH3 cells following FUS knockdown. The PSI value for exon 3 skipping was calculated with rMATS. **D** The positive correlation between MDM2 and FUS based on the PIT1-lineage PitNETs and normal pituitary (n = 40). **E** RT-PCR with exon-spanning primers was used to quantify the inclusion of MDM2 exon 3 in GH3 and MMQ cells after FUS knockdown (n = 3). **F** Expression of MDM2 splice isoforms was quantified by qPCR using isoform-specific probes (n = 3). **G** qRT-PCR analysis of mRNA for MDM2 in GH3 and MMQ after FUS knockdown (n = 3). **H** qRT-PCR utilizing intron- or exon-specific primers was performed to quantify the level of pre-mRNA and mRNA (n = 3). GAPDH was used for normalization. **I** RNA FISH probes for MDM2 exon 3 were used for detection in GH3 (n = 3). Scale bar = 25 μm. **J** RIP-qPCR was conducted to confirm the interaction between FUS protein and MDM2 pre-mRNA in GH3 and MMQ cells via anti-FUS antibody (n = 3). **K** RIP-qPCR was utilized to assess the binding affinities of FUS at its target sites following mutations of its functional domains in GH3 cells (n = 3). **L** qRT-PCR analysis of mRNA for MDM2 in GH3 cells following mutations of FUS functional domains (n = 3). **M** An MDM2 exon 3 minigene was constructed using the pSPL3 vector for the validation of alternative splicing, incorporating a mutation in the FUS binding sequence. RT-PCR with minigene primers was used to quantify the inclusion of MDM2 exon 3 (n = 3). Data are shown as mean ± SEM. *P < 0.05, **P < 0.01, ***P < 0.001.

To delineate the downstream effectors of FUS, we subsequently analyzed the functional profiles of all the candidate genes identified from the intersection of the datasets on differential alternative splicing and FUS-binding substrates (Figure 4A). For the candidate gene *MDM2*, IGV visualization of the sequencing alignments demonstrated significant FUS binding within both its exonic and intronic regions. Moreover, knockdown of FUS facilitated the skipping of exon 3 in the *MDM2* gene (Figure 4B-C). Analysis of primary PIT1-lineage PitNET transcriptomes consistently revealed a significant positive correlation between the expression levels of *FUS* and *MDM2* (Figure 4D). We subsequently designed primers spanning MDM2 exon 3 to validate the occurrence of alternative splicing events (Figure S3F). Analysis by semiquantitative PCR and fragment sizing confirmed that FUS knockdown promoted *MDM2* exon 3 exclusion across the GH3 and MMQ cells (Figure 4E). Changes in the expression levels of splice variants after FUS knockdown were confirmed by qPCR with isoform-specific probes (Figure 4F). To determine whether the degradation of aberrant splice variants is mediated by the nonsense-mediated mRNA decay (NMD) pathway, we knocked down the essential NMD protein UPF1 (Figure S3G). Following treatment with actinomycin D, compared with the controls, UPF1 knockdown prolonged the half-life of MDM2 ΔExon3 and decreased the PSI of MDM2 exon 3, demonstrating that MDM2 ΔExon3, rather than the full-length isoform, was regulated by NMD (Figure S3H). Degradation of aberrantly spliced isoforms via the NMD pathway may account for reduced MDM2 protein expression.

Mutually exclusive exon splicing events regulated by FUS were similarly validated through integrated RNA-seq and PCR analyses (Figure S3I-K). qPCR revealed that FUS knockdown markedly reduced mature MDM2 mRNA levels without affecting pre-mRNA abundance (Figure 4G-H). Moreover, fluorescence *in situ* hybridization (FISH) probes specifically targeting MDM2 exon 3 revealed a significant reduction in the number of transcripts containing exon 3 (Figure 4I).

To obtain further evidence of the direct binding of FUS to *MDM2* pre-mRNA, we designed PCR primers that target different regions of the pre-mRNA for RIP-qPCR. Notable FUS occupancy was detected in both the GH3 and MMQ cells, particularly across exon 3 and intron 3 (Figure 4J and Figure S3L). Previous research has revealed that RNA binding by the zinc finger (ZnF) and RNA recognition motif (RRM) domains underpins FUS-mediated splicing regulation 32. To analyze whether splicing activity depends on direct FUS-RNA interactions, we created functional domain mutants with four phenylalanine-to-leucine mutations in the RRM (F298L/F334L/F352L/F361L) or cysteine-to-alanine mutations in the ZnF (C428A/C444A/C447A) 33. Mutations within the RRM and ZnF domains impaired FUS binding to MDM2 pre-mRNA, concurrently eliminating its stimulatory influence on MDM2 expression (Figure 4K-L and Figure S3M). A pSPL3 minigene splicing assay was subsequently performed, as described in previous studies 34, to confirm the FUS-mediated inclusion of exon 3. Guided by the defined FUS-binding motif, four consensus sequences were identified within intron 3 of MDM2. Exon 3 of MDM2 and flanking introns were cloned and inserted into the pSPL3 minigene with a wild-type (WT) or mutant (Motif-Mut) sequence between the constitutive splice donor (SD) and splice acceptor (SA) sites. Mutation of the FUS-binding motif significantly disrupted MDM2 exon 3 inclusion in both the GH3 and MMQ cells (Figure 4M). Collectively, our findings identify MDM2 as a splicing substrate of FUS and demonstrate that FUS facilitates the expression of MDM2 by promoting the inclusion of exon 3.

### FUS facilitates the progression of PitNETs via the canonical MDM2-p53 signaling pathway

**Figure 5:**
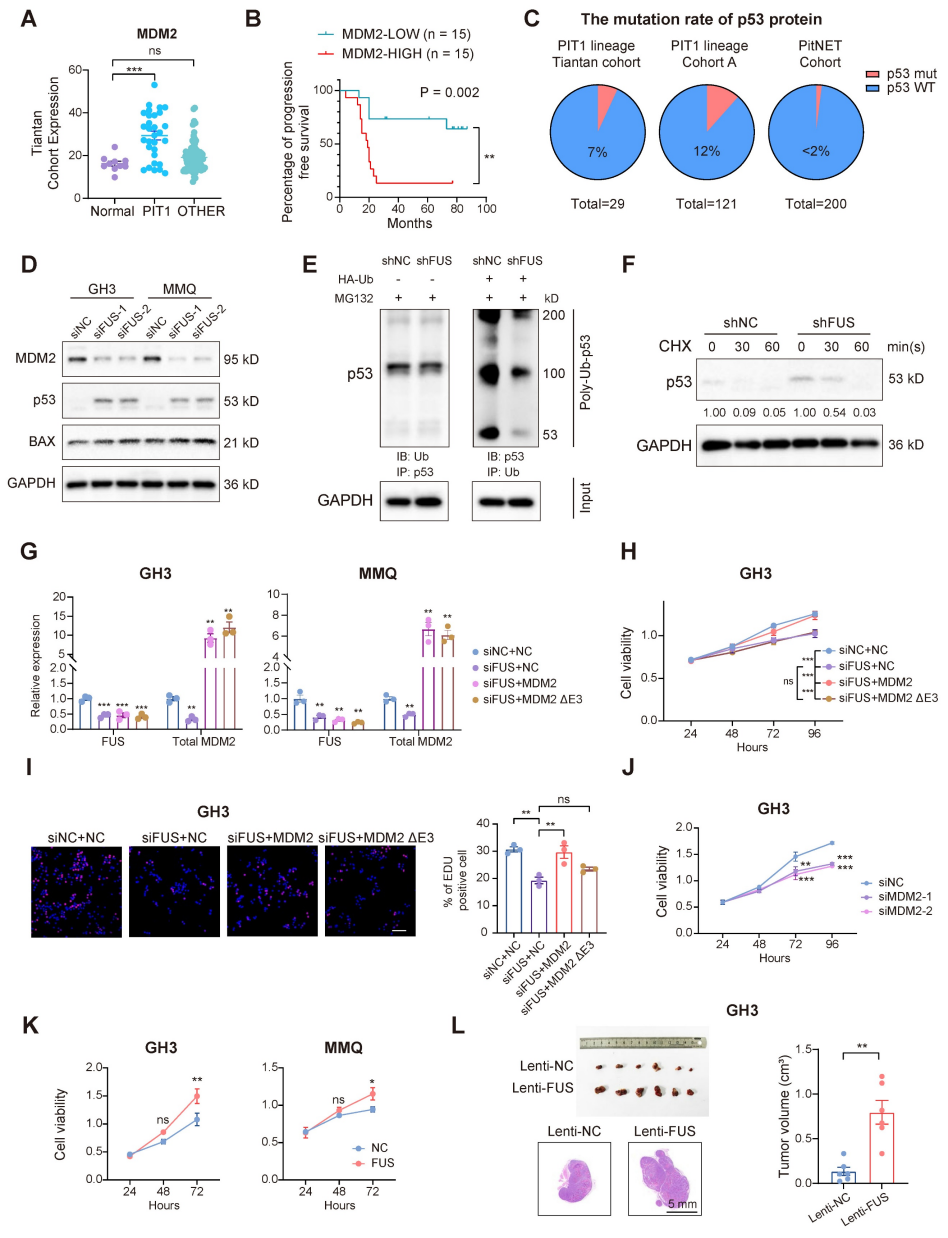
** FUS facilitates the progression of PitNETs via the canonical MDM2-p53 signaling pathway. A** FUS expression analysis in normal pituitary gland and different lineage of PitNETs based on our dataset (n = 125). **B** Kaplan-Meier analysis for patient PFS based on MDM2 expression in PIT1-lineage PitNETs based on our dataset (n = 30). **C** A comparative analysis of p53 mutation rates among different PitNET cohorts (n = 350). **D** Flow cytometry quantified cell cycle progression in GH3 and MMQ cells after PI staining and transfection with siNC, siFUS-1, or siFUS-2 (n = 3). **E** Western blot analysis for p53 ubiquitination in GH3 cells co-transfected with the plasmids encoding HA-ubiquitin (HA-Ub) or vector plasmids after MG132 treatment (n = 3). **F** Protein levels of p53 protein at the indicated time in GH3 cells after MG132 treatment (n = 3). **G** qRT-PCR analysis of FUS and MDM2 mRNA in GH3 and MMQ cells transfected with NC, MDM2, or MDM2 ΔE3 plasmids alongside FUS knockdown (n = 3). **H** Cell viability was measured by CCK-8 assay in GH3 and MMQ cells following co-transfection with NC, MDM2, or MDM2 ΔE3 overexpression plasmids, combined with siNC or siFUS (n = 3). **I** Cell proliferation was measured by EdU assay in GH3 for NC, MDM2, or MDM2 ΔE3 overexpression plasmid transfected with siNC or siFUS (n = 3). Scale bar = 100 μm. **J** Cell viability of GH3 after transfection with siNC, siMDM2-1 or siMDM2-2 (n = 3). **K** Cell viability of GH3- and MMQ-FUS-OE or NC (n = 3). **L** Representative tumor image and tumor volume of subcutaneous xenograft experiments of GH3 with FUS overexpression 3 weeks after tumor implantation (n = 6 mice per group). Representative images of HE staining of subcutaneous xenografts. Scale bar = 5 mm. Data are shown as mean ± SEM. *P < 0.05, **P < 0.01, ***P < 0.001.

To investigate whether the oncogenic function of FUS in PitNETs is mediated through its regulation of MDM2 splicing, we analyzed transcriptomic data and found that compared with that in normal pituitary tissue, MDM2 expression was markedly elevated in PIT1-lineage tumors (Figure 5A). Moreover, within our PIT1-lineage cohort or across all lineages, compared with patients with lower MDM2 expression, patients with elevated MDM2 expression demonstrated significantly reduced progression-free survival after surgery (Figure 5B and Figure S4A). Although MDM2 may have additional independent functions, it is consistently recognized as a negative regulator of the p53 tumor suppressor 35. We next systematically analyzed the frequency of p53 mutations across various PitNET cohorts. In both our in-house cohort and published studies, the incidence of p53 mutations remained consistently low, not surpassing 12% in either PIT1-lineage or all PitNETs (Figure 5C) 36,37. Whole-genome sequencing analyses of both the GH3 and MMQ cell lines revealed that intact, wild-type p53 functionality was preserved (Figure S4B). FUS knockdown markedly reduced MDM2 levels while simultaneously increasing the levels of p53 and its pro-apoptotic effector BAX (Figure 5D and Figure S4C). In contrast, MDM4, another protein involved in the regulation of p53, was neither transcriptionally modulated by FUS nor associated with PFS in PIT1-lineage PitNETs (Figure S4D-E). These findings suggest that MDM2 contributes to the progression of PIT1-lineage PitNETs by attenuating wild-type p53 activity. As expected, the knockdown of FUS expression significantly increased the poly-ubiquitination of p53 and reduced its half-life, indicating that MDM2 suppressed p53 activity through the canonical ubiquitin‒proteasome pathway (Figure 5E-F).

To determine the functional significance of FUS-mediated MDM2 splicing, we transfected GH3 and MMQ cells with splicing-independent overexpression constructs encoding either full-length MDM2 or MDM2 ΔExon3 (Figure 5G). The overexpression of full-length MDM2, rather than the ΔExon3 variant, partially reversed the proliferative defects induced by FUS knockdown (Figure 5H-I and Figure S4F). Moreover, MDM2 ΔExon3 overexpression did not reverse the BAX activation caused by FUS knockdown and thus failed to inhibit p53 function (Figure S4G). Moreover, individual knockdown of MDM2 was also sufficient to suppress cell viability and upregulate BAX expression without affecting Tp53 mRNA expression (Figure 5J and Figure S4H-L).

FUS overexpression concurrently upregulated MDM2 and PIT1 expression and significantly increased the viability of GH3 and MMQ cells (Figure 5K and Figure S5A-C). In the *in vivo* experiments, FUS overexpression led to an increase in the volume of subcutaneous xenograft tumors (n = 6/group), which was accompanied by elevated Ki67 indices and increased staining intensity of MDM2 (Figure 5L and Figure S5D-G). These findings indicate that FUS promotes the progression of PIT1-lineage PitNETs by modulating the canonical MDM2-p53 pathway through splicing regulation.

### ASO-mediated MDM2 exon skipping exhibits potent anti-PitNET efficacy

**Figure 6:**
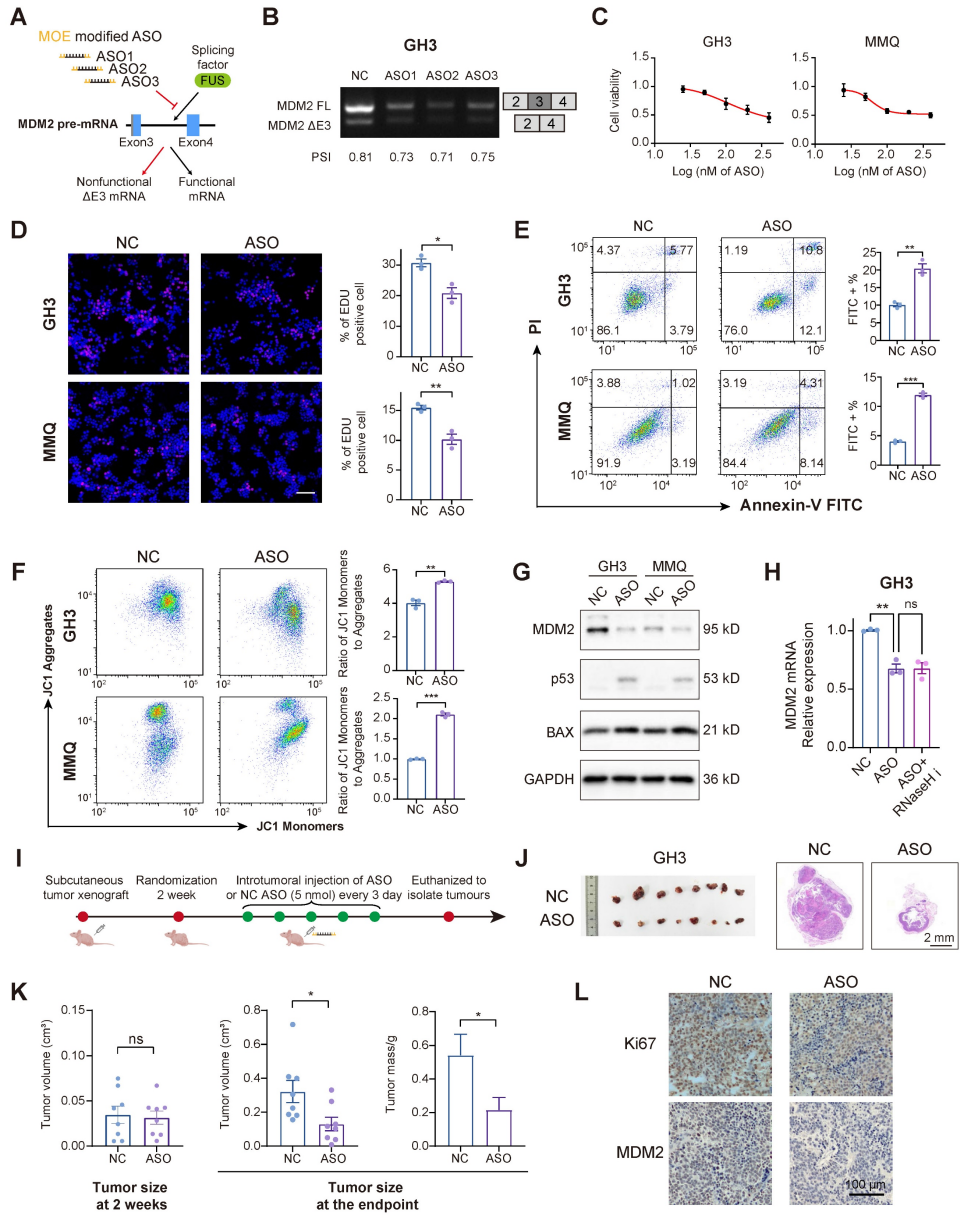
** Splice-switching ASOs against MDM2 exon 3 demonstrate antitumor efficacy. A** The schematic diagram illustrating the design of MOE modified ASOs targeting the AS of MDM2. **B** RT-PCR with exon-spanning primers was used to quantify the inclusion of MDM2 exon 3 in GH3 cells after three ASOs (100 nM) for 48 h. **C** Cell viability was measured by CCK-8 assay in GH3 and MMQ after 100 nM ASOs treatment for 48 h (n = 3). **D** Cell proliferation was measured by EdU assay in GH3 after 100 nM ASOs treatment for 48 h (n = 3). Scale bar = 100 μm. **E** Apoptosis was quantified by Annexin V-FITC/PI flow cytometry in GH3 and MMQ cells after 100 nM ASOs treatment for 48 h (n = 3). **F** JC-1 labeling and flow cytometry assessed mitochondrial membrane potential in GH3 and MMQ cells after 100 nM ASOs treatment for 48 h (n = 3). **G** Protein level of MDM2, p53 and BAX of GH3 and MMQ after 100 nM ASOs treatment for 48 h (n = 3). **H** mRNA level of MDM2 in GH3 after 100 nM ASOs treatment for 48 h with or without RNaseH inhibitor compound lA-6 (n = 3). **I** The flowchart illustrating the administration of ASO treatment in subcutaneous xenograft models. **J** Representative tumor image and HE staining of subcutaneous xenograft experiments of GH3 cells with intratumoral injection of ASO or ASO-NC. (n = 8 mice per group). Scale bar = 2 mm. **K** Tumor volume and tumor weight of subcutaneous xenograft experiments of GH3 after intratumoral injection of ASO or ASO-NC. **L** IHC for Ki67 and MDM2 levels of subcutaneous xenograft. Scale bar = 100 μm. Data are shown as mean ± SEM. *P < 0.05, **P < 0.01, ***P < 0.001.

ASOs can bind to pre-mRNAs, facilitating splice-switching or promoting RNA degradation. These mechanisms have been investigated as therapeutic strategies for various diseases associated with splicing abnormalities 38. To assess the therapeutic potential of ASOs in PitNETs, we designed 3 ASOs that target MDM2 pre-mRNA, incorporating 2′-O-methoxyethyl (MOE) modifications to increase their affinity and stability (Figure 6A and Figure S6A). All three ASOs successfully induced skipping of MDM2 exon 3, with ASO2 exhibiting the highest splice-switching efficiency (Figure 6B). ASO2 was selected for further experiments, as treatment at a concentration of 100 nM for a duration of 48 hours effectively inhibited the proliferation of both GH3 and MMQ cells (Figure 6C-D). In alignment with the previous FUS-knockdown treatment, the administration of ASOs similarly promoted apoptosis and attenuated mitochondrial activity (Figure 6E-F). Mechanistically, ASO treatment markedly reduced the MDM2 protein level, which in turn decreased p53 expression and elevated the expression of its pro-apoptotic downstream protein BAX (Figure 6G and Figure S6B-C). Notably, cotreatment with an RNase H inhibitor failed to restore MDM2 mRNA levels 39, demonstrating that the ASO modulates splicing rather than directly promoting transcript degradation (Figure 6H and Figure S6D).

We extended our investigation to an *in vivo* model to validate the tumor-suppressive effects of ASO in PitNETs. Utilizing GH3 cells, we developed a subcutaneous xenograft model and administered an ASO via intratumoral injection (Figure 6I). ASO treatment significantly reduced tumor volume (Figure 6J-K). IHC analysis demonstrated that ASO inhibited tumor proliferation, reduced MDM2 expression, and restored the expression levels of both p53 and BAX (Figure 6L and Figure S6E-F).

**Figure 7:**
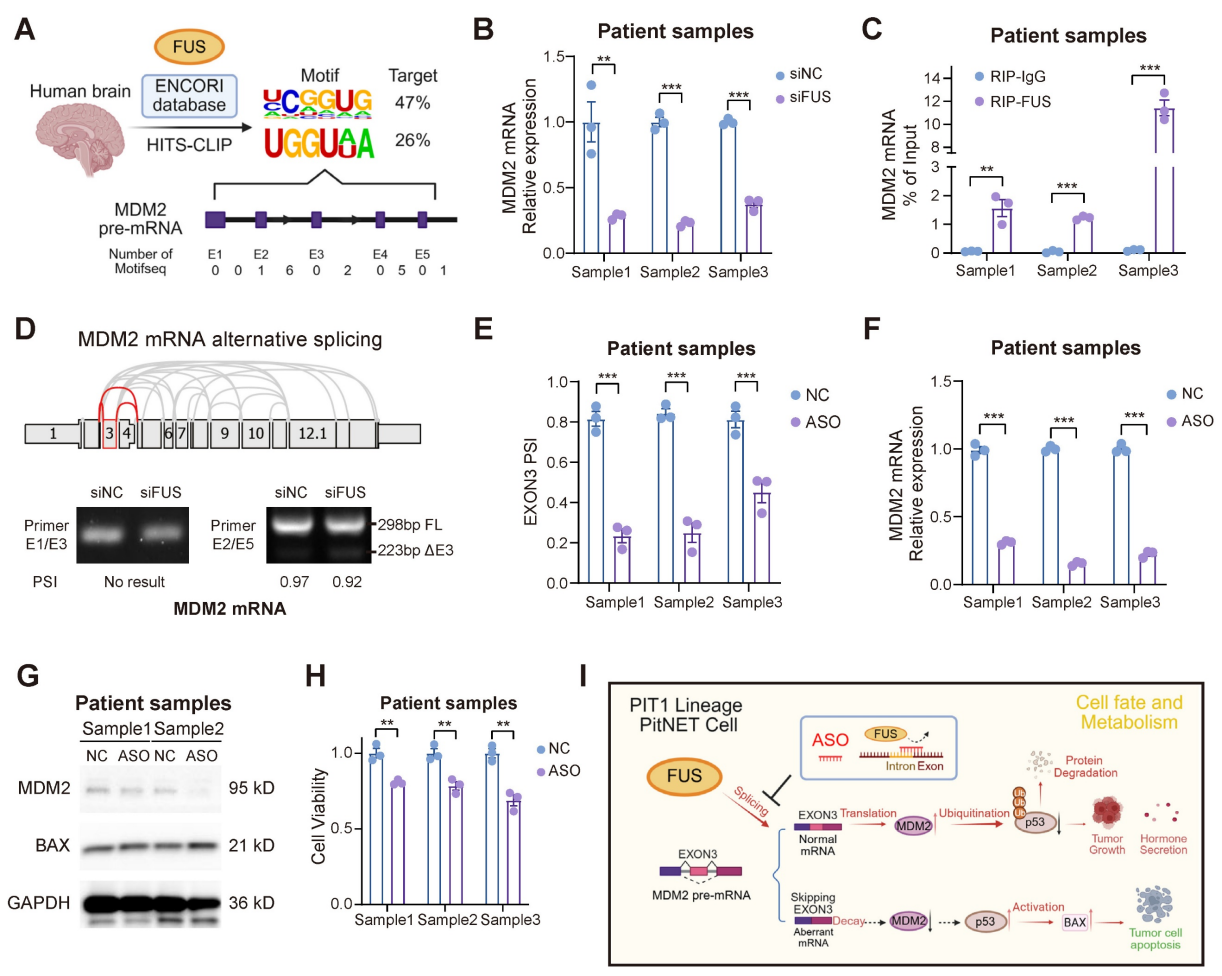
** Applications of ASO therapy in primary PIT1-lineage PitNETs. A** Exploring the FUS binding motif with MDM2 pre-mRNA across human brain regions utilizing the ENCORI database. **B** PCR analysis of mRNA for MDM2 in primary PIT1-lineage samples with siNC or siFUS (n = 3). **C** RIP-qPCR was conducted to confirm the interaction between FUS protein and MDM2 pre-mRNA in primary PIT1-lineage samples via anti-FUS antibody (n = 3). **D** Screening of MDM2 AS events in TCGA database and RT-PCR with exon-spanning primers quantify the inclusion of MDM2 exon 3 in primary PIT1-lineage samples after FUS knockdown (n = 3). **E** Quantification of splice isoform ratios utilizing isoform-specific qPCR in primary PIT1-lineage samples after 100 nM ASOs treatment for 48 h (n = 3). **F** PCR analysis of mRNA for MDM2 in primary PIT1-lineage samples after 100 nM ASOs treatment for 48 h (n = 3). GAPDH was used for normalization. **G** Western blot analysis for MDM2 and BAX of primary PIT1-lineage samples after 100 nM ASOs treatment for 48 h (n = 3). **H** Cell viability was measured by CCK-8 assay in primary PIT1-lineage samples after 100 nM ASOs treatment for 48 h (n = 3). **I** Schematic of ASO-directed blockade of FUS-mediated alternative splicing of MDM2 exon 3 to activate p53-dependent apoptosis. Data are shown as mean ± SEM. *P < 0.05, **P < 0.01, ***P < 0.001.

To further substantiate the therapeutic potential of ASO treatment, we expanded our evaluation to primary PIT1-lineage PitNET cells. We initially screened the HIS-CLIP dataset in the ENCORI database to identify the FUS binding motif in human brain cells (Figure 7A). Primary tumor cells of the PIT1 lineage expressing wild-type p53 were isolated and subsequently subjected to validation (Table S7). FUS not only regulated the abundance of *MDM2* mRNA but also directly interacted with the mRNA through direct binding in primary cells (Figure 7B-C). Guided by the comprehensive MDM2 splicing landscape in The Cancer Genome Atlas (TCGA) SpliceSeq, we designed primers spanning multiple exon-intron boundaries to investigate splice variants. Our findings demonstrate that the skipping of MDM2 exon 3 is evolutionarily conserved (Figure 7D). rMATS analysis of the PIT1-lineage PitNETs revealed a reduced inclusion rate of exon 3 in the low-FUS group. Nevertheless, the limited sample size resulted in the difference not reaching statistical significance (Figure S7A).

Pancancer TCGA database analysis revealed MDM2 upregulation in the majority of tumor types compared with their corresponding normal tissues (Figure S7B). We subsequently designed and evaluated 3 ASO sequences based on the human FUS motif in primary PitNET cells, and ASO showed enhanced efficacy in suppressing FUS-mediated splicing events (Figure 7E and Figure S7C-D). Through splice-switching, ASO treatment significantly downregulated MDM2 expression while simultaneously upregulating the expression of the pro-apoptotic protein BAX (Figure 7F-G and Figure S7E). ASO treatment suppressed the proliferation of primary cells (Figure 7H), underscoring its potential as a promising clinical strategy for PIT1-lineage PitNETs.

Additionally, we assessed the therapeutic efficacy of established PIT1-lineage agents, octreotide and cabergoline, in addition to the MDM2 inhibitor idasanutlin, against PIT1-lineage cells. After 48 hours of treatment at a concentration of 10 μM, octreotide, cabergoline, and idasanutlin demonstrated significant efficacy in the GH3 and MMQ cell lines; notably, combined administration potentiated this effect (Figure S7F). However, the three drugs were largely ineffective against primary cells at a concentration of 10 μM, possibly due to clinical drug resistance in many surgical patients (Figure S7G). Dose‒response assays revealed an IC50 of approximately 15 μM for idasanutlin in primary PIT1-lineage PitNET cells, limiting the clinical translation of MDM2 inhibitors (Figure S7H). Considering that ASO treatment maintained substantial effectiveness, this difference highlights the potential therapeutic application of targeting the FUS-MDM2 axis via ASO in PIT1-lineage PitNETs.

## Discussion

Previous research has demonstrated the high prevalence of splicing abnormalities in PitNETs. For example, splicing dysregulation in the T-PIT lineage is driven by alterations in ESRP1, which are correlated with poorer clinical outcomes 14. In this study, based on the expression analysis and functional screening of PIT1-lineage PitNETs, we focused on the expression of the splicing factor FUS and revealed that its expression level was correlated with postoperative hypopituitarism. Aberrant splicing mediated by FUS is a crucial mechanism underlying neurodegenerative diseases, and dysregulated FUS also contributes to breast cancer progression 40,41.

Our research demonstrated that FUS facilitates the proliferation of tumor cells and xenograft tumor growth of PIT1-lineage PitNETs. Moreover, the inhibition of FUS leads to the activation of the p53 pathway and an increase in apoptosis. These findings underscore the critical tumor-promoting role of FUS in PitNETs. In addition, as demonstrated by both our data and the CGGA-CNS database, FUS upregulation is not restricted to the PIT1 lineage, suggesting a potentially universal role for FUS across PitNET lineages. Nevertheless, given the intrinsic differences among various lineages, this hypothesis requires further validation.

In this study, we systematically analyzed the splicing landscape regulated by FUS through transcriptome and RIP-seq analyses. Our findings indicate that exon skipping is the predominant splicing event associated with FUS knockdown. Through functional analysis and alternative splicing assays, we confirmed that MDM2 serves as a downstream splicing substrate of FUS in PitNETs. Aberrant splicing of exon 3 in MDM2 has been reported to regulate neurodevelopment and tumorigenesis by modulating the p53 pathway 42,43. Our experiments further demonstrated that the splicing regulation of exon 3 in MDM2 is a widely occurring splicing regulatory mechanism that promotes tumor cell proliferation and inhibits apoptosis in PitNETs. Although FUS can also regulate MDM2 levels via ubiquitination, our experiments revealed that FUS does not directly interact with MDM2 in PitNETs 44. Our results demonstrated that FUS performs a splicing function and promotes MDM2 expression through direct binding to pre-mRNA.

The incidence of p53 mutations in PitNETs is relatively low; however, p53 mutations are associated with a poor prognosis 45,29. MDM2 modulates the proliferation and apoptosis of PitNETs via the canonical p53 signaling pathway in the SF1 and T-PIT lineages 30,46. Our experiments demonstrated that MDM2 is an independent prognostic factor for tumor recurrence and further confirmed that MDM2 also modulates the activity of wild-type p53 and its associated apoptotic pathways in the PIT1 lineage. More importantly, we elucidated the mechanisms underlying the dysregulation of the MDM2-p53 pathway in PitNETs. However, the reasons for the aberrant upregulation of FUS splicing factors still require further investigation.

RNA-based strategies are rapidly advancing, with the therapeutic potential of ASOs in intracranial tumors being extensively investigated 20,47. The splicing factor SRSF3 drives the inclusion of exon 6 in MDM4, whereas ASO-mediated skipping of exon 6 effectively reduces MDM4 levels and significantly inhibits melanoma growth 48. In this study, we designed ASOs targeting the binding sites of FUS within the pre-mRNA of MDM2. These ASOs effectively reduced MDM2 mRNA levels through splice switching and subsequently induced apoptosis in PIT1-lineage PitNETs. Through experiments involving both xenograft and primary tumor cells, we validated the efficacy of ASOs and once again confirmed the universality of the MDM2-p53 axis in cancer therapy. Our findings suggest that ASO-mediated skipping of MDM2 exon 3 provides a novel treatment for PitNETs (Figure 7I).

Our study established a mechanistic role for FUS in regulating MDM2 splicing and PIT1-lineage PitNET progression; however, we acknowledge important limitations in exploring these findings in cell line models of human disease. First, species-specific differences in splicing regulation and tumor microenvironments may limit direct translation. Although the FUS-MDM2-P53 axis is evolutionarily conserved, subtle variations in splice site recognition or protein‒protein interactions between rat and human systems could result in different phenotypes. Second, cell lines—whether rat cells or primary cells—lack the three-dimensional architecture, cellular heterogeneity, and stromal interactions present in primary PitNETs, potentially oversimplifying the therapeutic responses observed. However, several lines of evidence support the clinical relevance of our findings. The aberrant splicing patterns and FUS-dependent phenotypes we identified in a rat model were recapitulated in human PIT1-lineage PitNET specimens. Moreover, the ASO targeting strategy demonstrated comparable efficacy in primary PIT1-lineage cells, suggesting mechanistic conservation. Future studies should also assess whether species-specific differences in pharmacokinetics and ASO delivery efficiency influence treatment outcomes. Ultimately, our model provided critical mechanistic insights and proof-of-concept for targeting FUS-mediated splicing; however, the findings should be interpreted as a foundation for—rather than as a substitute for—human translational studies.

## Supplementary Material

Supplementary figures and tables.

## Abbreviations

PitNETs: pituitary neuroendocrine tumors
NBTRC: National Brain Tumor Registry of China
pre-mRNA: precursor messenger RNA
TCGA: The Cancer Genome Atlas
RIP-seq: RNA immunoprecipitation sequencing
ASOs: antisense oligonucleotides
RBPs: RNA binding proteins
AS: Alternative splicing
GO: Gene Ontology
MOE: 2′-O-methoxyethyl
ZnF: zinc finger
GSEA: Gene set enrichment analysis
IHC: Immunohistochemistry
FISH: Fluorescence *in situ* hybridization
RIP: RNA immunoprecipitation
NMD: nonsense-mediated mRNA decay
SD: splice donor
SA: splice acceptor
IC50: half maximal inhibitory concentration
