# Supplementary material for: Splicing factor FUS facilitates the progression of PIT1-lineage PitNETs by upregulating MDM2
Source: Theranostics. 2026 Jan 1;16(6):3032–49. doi: 10.7150/thno.124068 (PMC12775928; doi:10.7150/thno.124068)
Supplement: Supplementary file 1 — Supplementary figures and tables. [file thnov16p3032s1.pdf]

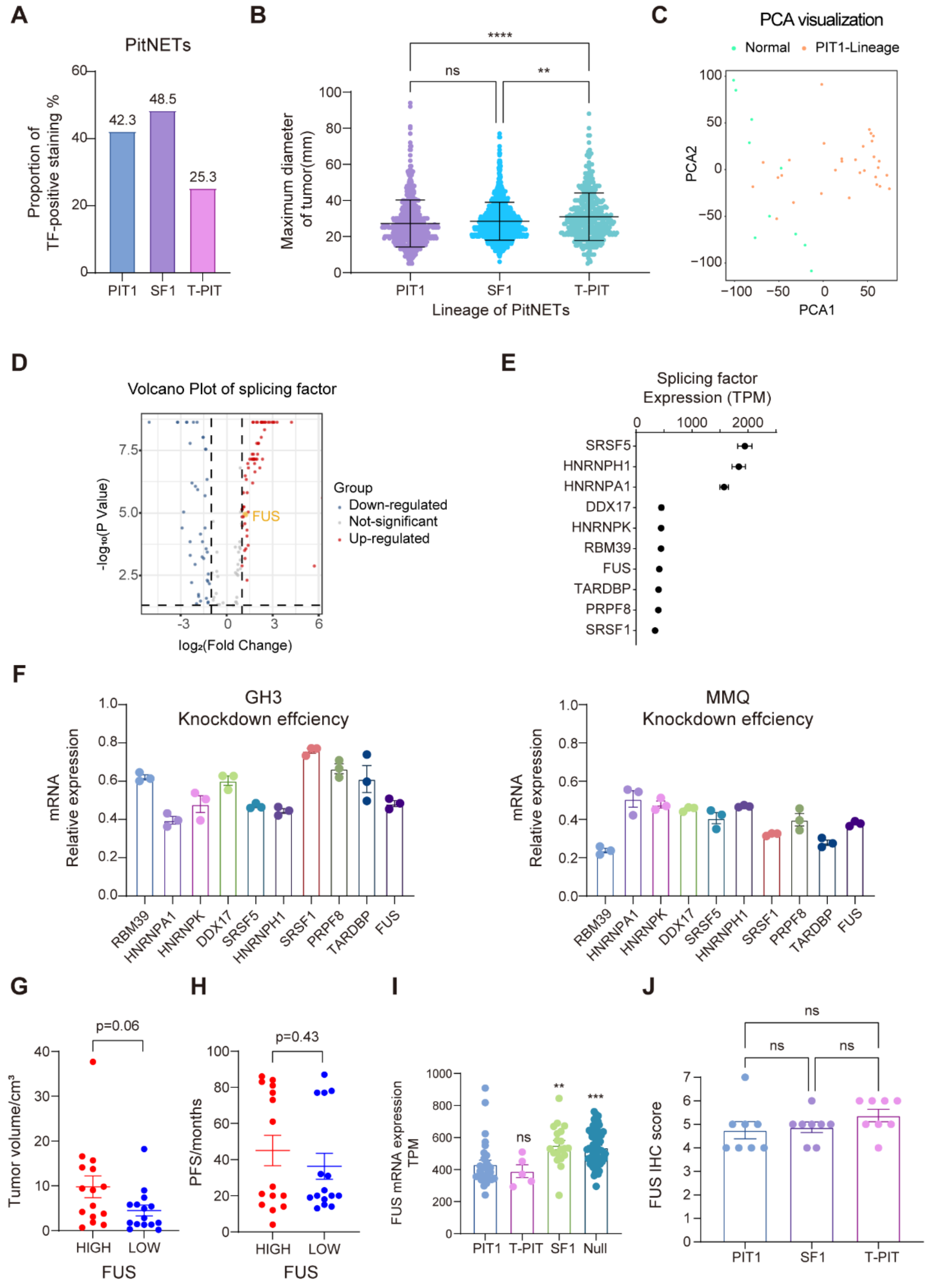

Figure S1. Lineage distribution and splicing-factor expression profiles of PitNETs

A Proportion of PitNET samples positive for lineage-specific transcription factors, including mixed phenotypes (n = 3717).

B Tumor size across the three PitNETs lineages in the NBTRC cohort (n = 571, 770, and 438, respectively).

C PCA visualization of sequencing data from normal and PIT1-lineage samples.
D Volcano plots of splicing factors differentially expressed in PIT1-lineage samples. FUS is highlighted in orange.
E Top 10 splicing factors ranked by mRNA abundance based on transcriptome in PIT-1 lineage PitNETs.
F qRT-PCR analysis of mRNA expression of splicing factors in GH3 and MMQ cell lines transfected with siRNA (n = 3).
G Tumor size comparison between FUS-high and FUS-low groups in PIT-1 lineage PitNETs (n = 30).
H PFS comparison between FUS-high and FUS-low groups in PIT-1 lineage PitNETs (n = 30).
I FUS mRNA expression across PitNET lineages by RNA-seq.
J IHC scoring of FUS in different lineage of PitNETs (n = 8 per group). Data are shown as mean ± SEM. *P < 0.05, **P < 0.01, ***P < 0.001.

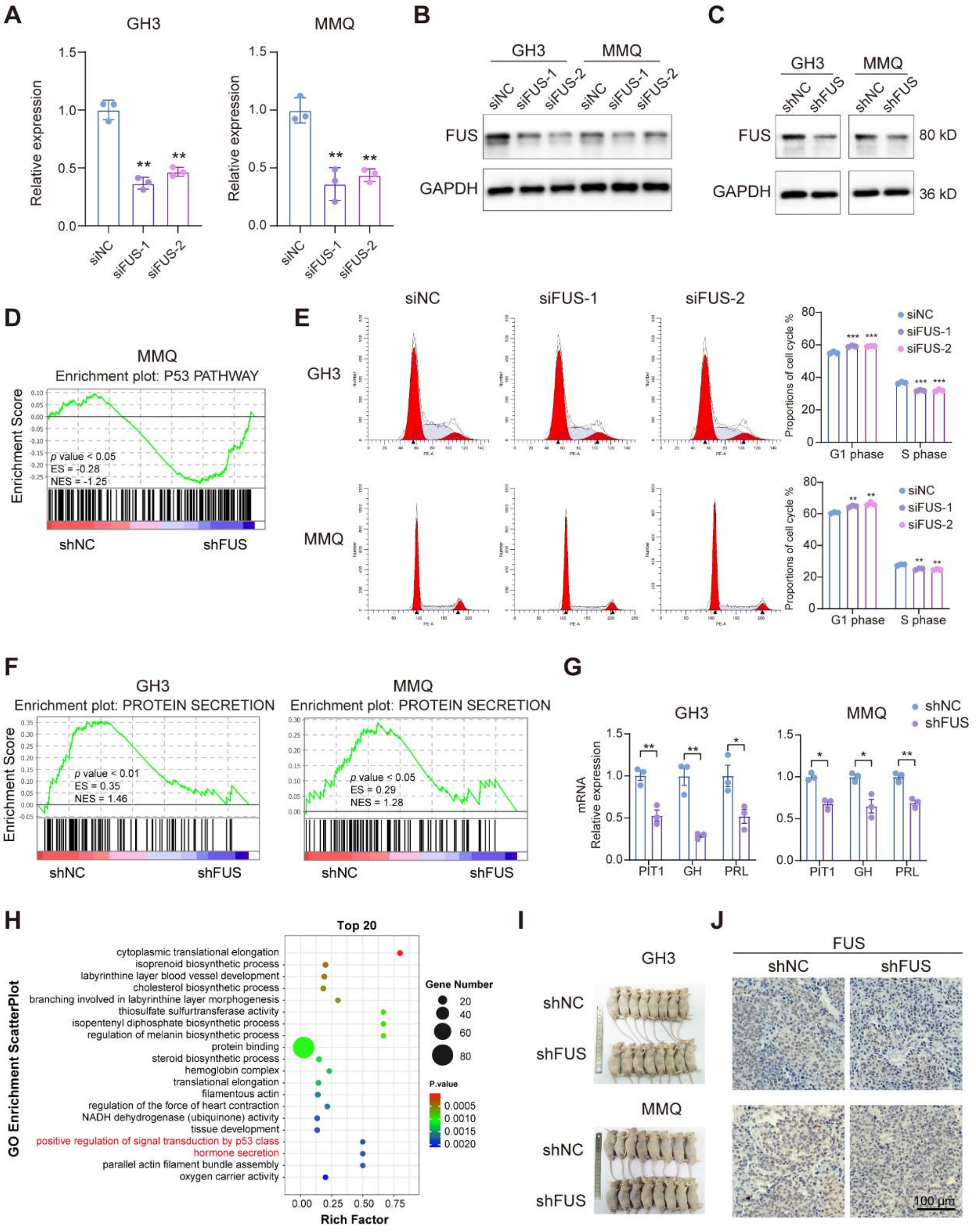

Figure S2. FUS knockdown activates the p53 pathway and suppresses hormone secretion in PIT-1 lineage cell lines.

A qRT-PCR analysis of FUS expression in GH3 and MMQ cell lines transfected with siNC, siFUS-1 and siFUS-2 (n = 3).

B Protein levels of FUS were assessed in GH3 and MMQ cells following transfection with control siRNA or siFUS (n = 3).

C Protein levels of FUS were assessed in GH3- and MMQ-shNC or -shFUS (n = 3).

D p53 pathway in GSEA analysis from MMQ cells after FUS knockdown.

E Flow cytometry for cell cycle analysis of PI stained GH3 and MMQ transfected

with control siRNA or siFUS (n = 3).

F Protein secretion pathway in GSEA analysis from GH3 and MMQ cells after FUS knockdown.

G qRT-PCR analysis of PIT1, GH and PRL expression in GH3 and MMQ cell lines transfected with shNC or shFUS (n = 3).

H GO enrichment analysis of differentially expressed genes in GH3 cells following FUS knockdown.

I Representative images of xenograft mice bearing subcutaneous tumor derived from GH3 and MMQ cells, transfected with shNC or shFUS (n = 8 mice per group).

J IHC for FUS of subcutaneous xenograft derived from GH3 and MMQ cells, transfected with shNC or shFUS. Scale bar = 100 μm. Data are shown as mean ± SEM. *P < 0.05, **P < 0.01, ***P < 0.001.

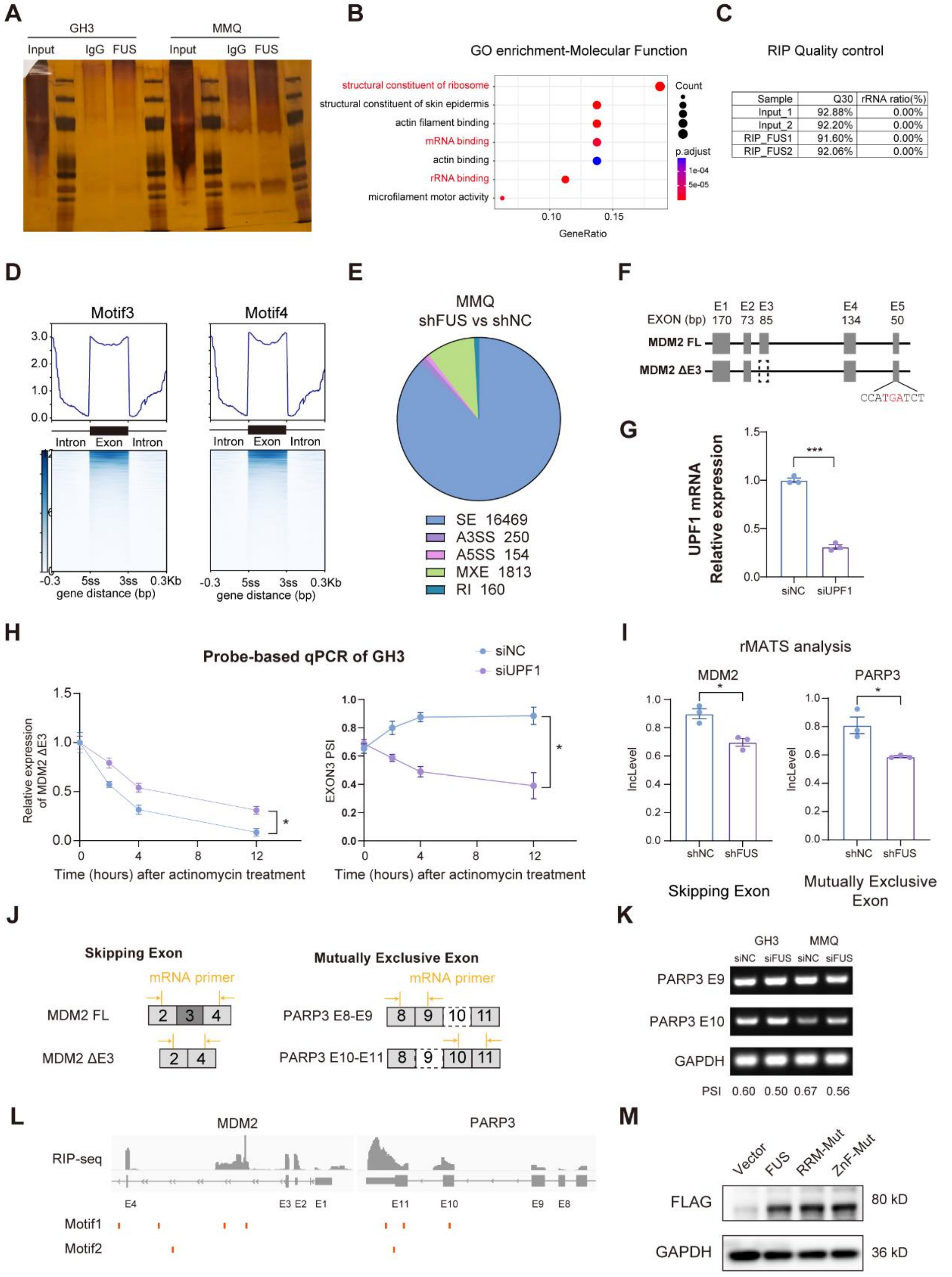

Figure S3. FUS regulates alternative splicing by directly binding to pre-mRNA.

A Silver staining of SDS-PAGE to detect proteins associated with FUS protein in GH3 and MMQ by immunoprecipitation.

B GO enrichment analysis of FUS-binding proteins in GH3 and MMQ cells followed by mass spectrometry.

C Quality control of Input and RIP samples.

D Heat map showing FUS-binding motif distribution in the exon region and 300 bp around the 3' or 5' splice site junction, generated using DeepTools. Exon lengths were normalized to 300 bp.
E Pie chart showing the distribution of various AS types in transcriptome data derived from MMQ cells following FUS knockdown.
F Diagram illustrating the structure of MDM2 exons and the premature termination codon resulting from EXON3 deletion.
G Expression of UPF1 in GH3 cells treated with siNC or siUPF1 (n = 3).
H Expression of MDM2 ΔE3 and changes of splice isoform ratio following actinomycin D treatment in GH3 cells transfected with siNC or siUPF1 (n = 3).
I Statistics of representative alternative splicing events and their inclusion-level derived from transcriptomic data analyzed using rMATS.
J The schematic representation of exon-spanning primers designed for AS detection.
K RT-PCR with exon-spanning primers was used to quantify the inclusion of PARP3 exon 9 and 10 in GH3 after FUS knockdown (n = 3).
L FUS binding motifs around the regulated exon 3 of MDM2 and exon8/9 of PARP3 utilizing RIP-seq.
M Western blot to detect constructs expression encoding FLAG-tagged wild-type FUS and its relevant domain mutants (n = 3). Data are shown as mean ± SEM. *P < 0.05, ***P < 0.001.

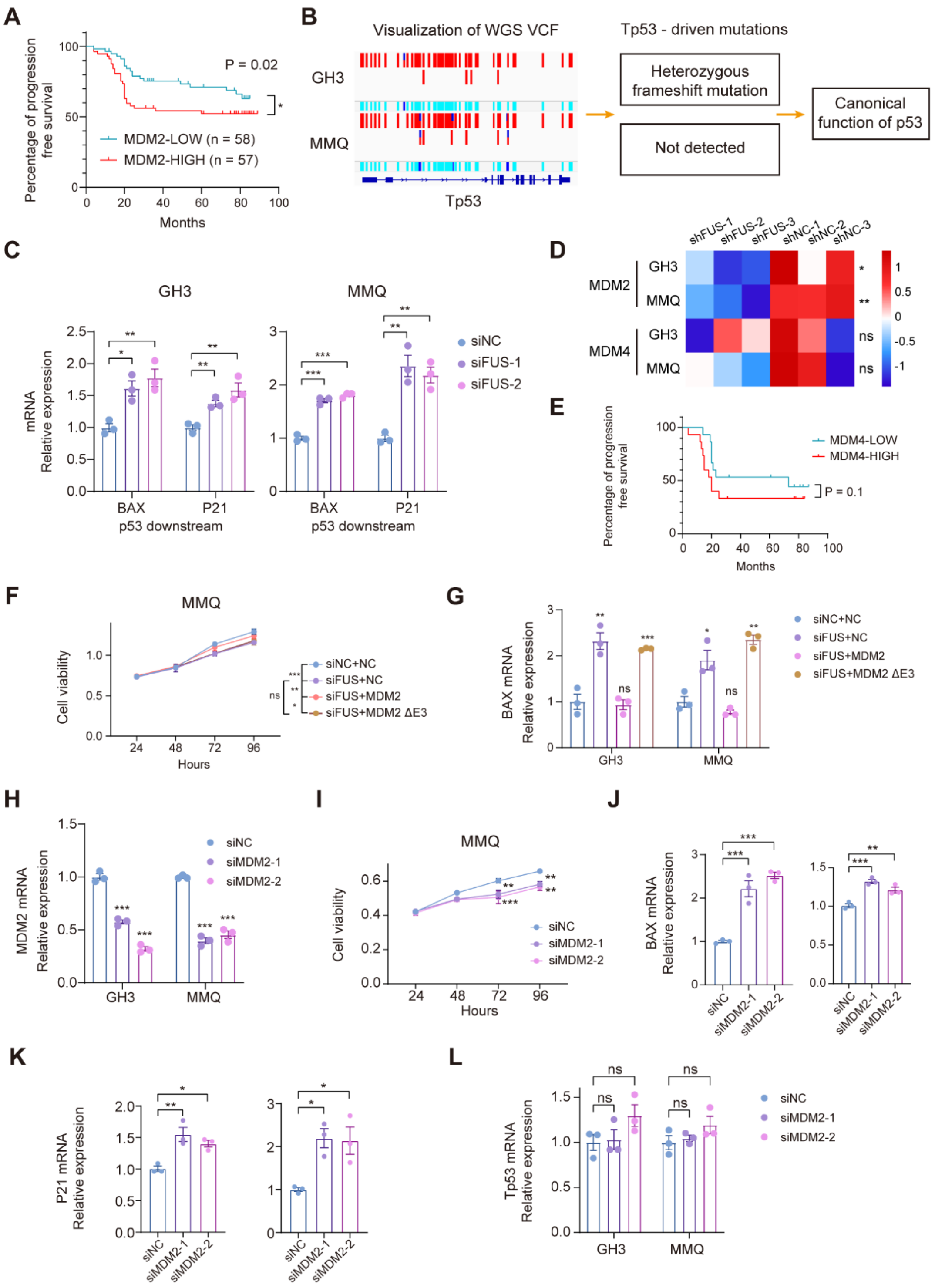

Figure S4. The MDM2–p53 axis regulated by FUS, represents a crucial pathway in PIT1-lineage PitNETs.

A Kaplan–Meier analysis for patient PFS based on MDM2 expression across all PitNETs based on our dataset (n = 115).

B Visualization of the p53 mutational landscape utilizing VCF data of GH3 and MMQ cells.

C qRT-PCR analysis of mRNA level of p53 downstream BAX and P21 in GH3

and MMQ after FUS knockdown (n = 3). GAPDH was used for normalization.

D Heatmap displaying MDM2 and MDM4 expression obtained from transcriptome for GH3 and MMQ transfected with shNC and shFUS.

E Kaplan–Meier analysis for patient PFS based on MDM4 expression in PIT1 lineage PitNETs based on our dataset (n = 30).

F The CCK-8 assay assessed cell viability in GH3 and MMQ cells transfected with NC, MDM2, or MDM2 ΔE3 overexpression plasmids, combined with siNC or siFUS (n = 3).

G BAX expression of GH3 and MMQ cells transfected with NC, MDM2, or MDM2 ΔE3 overexpression plasmids, combined with siNC or siFUS (n = 3).

H qRT-PCR analysis of MDM2 expression in GH3 and MMQ cell lines transfected with siNC, siMDM2-1 and siMDM2-2 (n = 3).

I Cell viability was measured by CCK-8 assay in MMQ after transfection with siNC, siMDM2-1 or siMDM2-2 (n = 3).

J mRNA levels of BAX in MMQ after transfection with siNC, siMDM2-1 or siMDM2-2 (n = 3).

K mRNA levels of P21 in GH3 and MMQ after transfection with siNC, siMDM2-1 or siMDM2-2 (n = 3).

L mRNA levels of Tp53 in GH3 and MMQ after transfection with siNC, siMDM2-1 or siMDM2-2 (n = 3). Data are shown as mean ± SEM. *P < 0.05, **P < 0.01, ***P < 0.001.

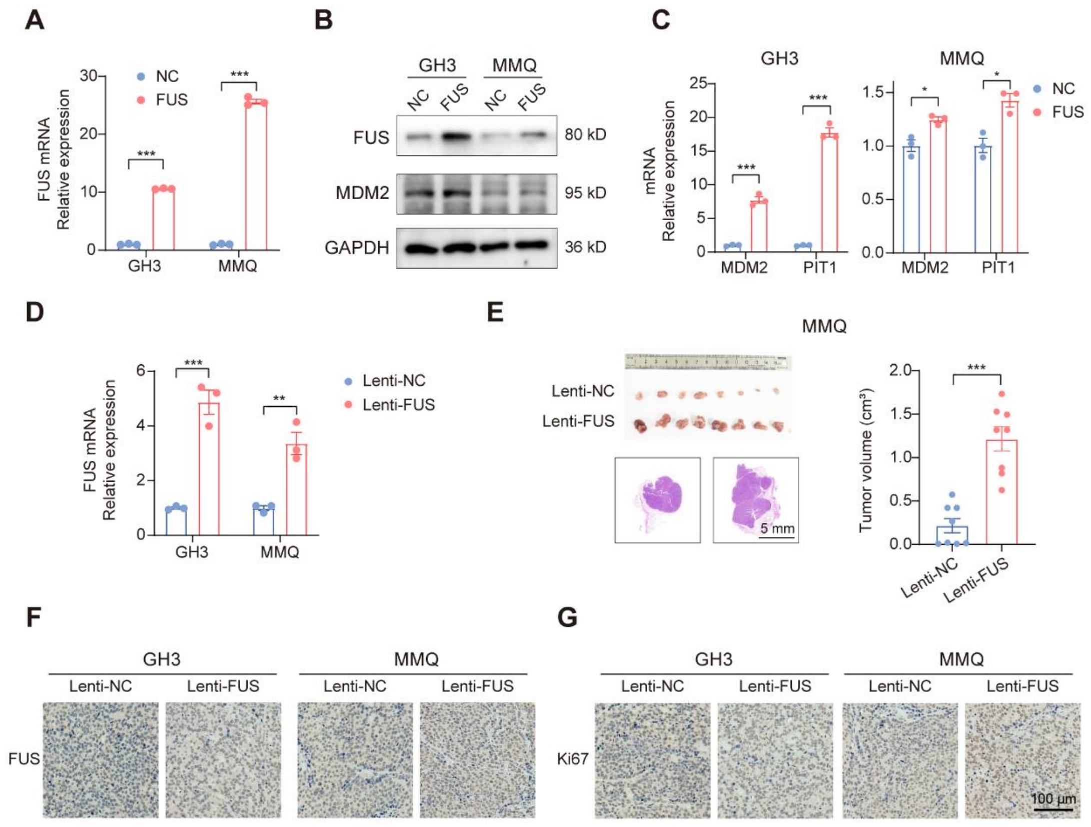

Figure S5. Overexpression of FUS promotes PIT1-lineage PitNET progression.

A FUS mRNA expression in GH3 and MMQ cells after transfection with FUS-overexpressing plasmid or negative control (n = 3).

B Protein level of FUS and MDM2 in GH3 and MMQ-FUS-OE or NC (n = 3).

C qRT-PCR analysis of MDM2 and PIT1 expression in GH3 and MMQ transfected with plasmid expressing FUS or NC (n = 3).

D FUS mRNA expression in GH3 and MMQ cells after lentiviral transduction with Lenti-FUS or Lenti-NC (n = 3).

E Representative tumor image and tumor volume of subcutaneous xenograft experiments of MMQ with FUS overexpression 3 weeks after tumor implantation (n = 8 mice per group). Representative images of HE staining of subcutaneous xenografts. Scale bar = 5 mm.

F-G IHC for FUS and Ki67 of subcutaneous xenograft derived from GH3 and MMQ cells, transfected with FUS or NC. Scale bar = 100 μm. Data are shown as mean ± SEM. *P < 0.05, **P < 0.01, ***P < 0.001.

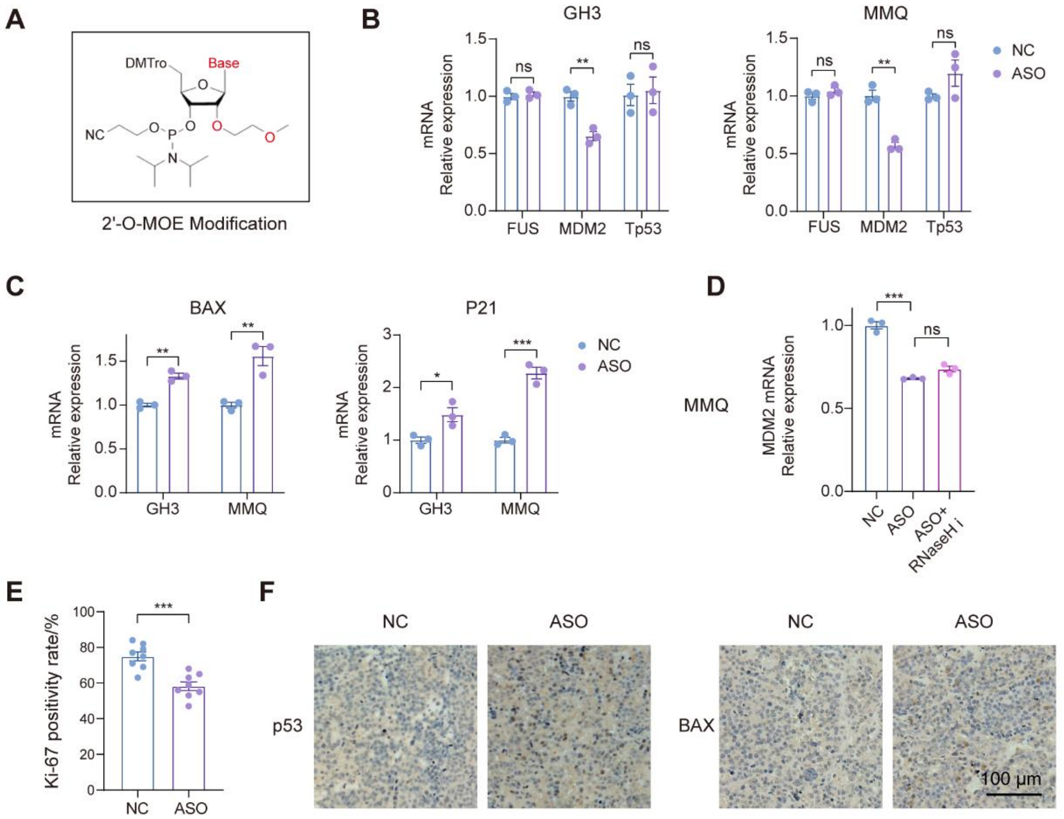

Figure S6. ASO-driven splicing switch restores p53 pathway activity in PIT1-lineage PitNETs.

A Structural diagram of ASO featuring 2′-O-methoxyethyl (MOE) modifications at both ends.

B mRNA levels of FUS, MDM2 and Tp53 in GH3 and MMQ after 100 nM ASOs treatment for 48 h (n = 3).

C mRNA levels of BAX and P21 in GH3 and MMQ after 100 nM ASOs treatment for 48 h (n = 3).

D mRNA levels of MDM2 in MMQ after 100 nM ASOs treatment for 48 h with or without RNaseH inhibitor compound IA-6 (n = 3).

E Quantitative Ki67 immunostaining analysis of subcutaneous xenografts treated with ASO or ASO-NC (n = 8).

F IHC for p53 and BAX of subcutaneous xenograft derived from GH3 after intratumoral injection of ASO or ASO-NC. Scale bar = 100 μm. Data are shown as mean ± SEM. *P < 0.05, **P < 0.01, ***P < 0.001.

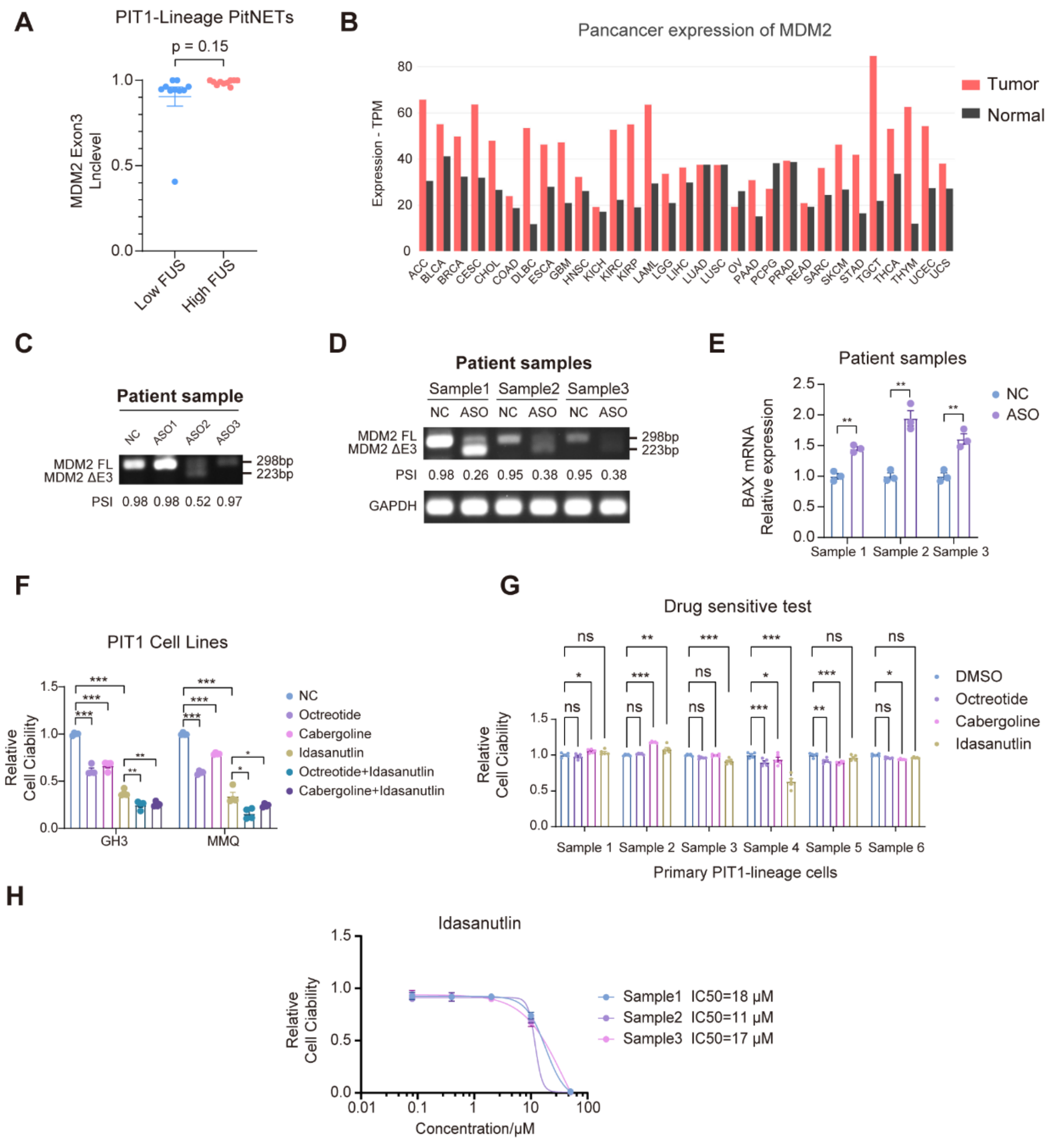

Figure S7. In PIT1-lineage PitNETs, splice-switching ASOs targeting MDM2 outperform conventional therapeutics.

A Comparison of the Inclevel of MDM2 exon 3 between FUS-high and FUS-low groups in PIT-1 lineage PitNETs (n = 30).

B MDM2 pan-cancer expression based on TCGA database.

C-D RT-PCR with exon-spanning primers quantify the inclusion of MDM2 exon 3 in primary PIT1-lineage sample following treatment with 3 different ASOs at a concentration of 100 nM for 48 hours (n = 3). ASO2 was selected for subsequent validation.

E mRNA levels of BAX in primary PIT1-lineage samples after 100 nM ASOs treatment for 48 h (n = 3).

F Comparative analysis of the efficacy of established PIT1-lineage drugs and the MDM2 inhibitor Idasanutlin in PIT1 cell lines following a 48-hour treatment at a concentration of 10 μM. The effects of the drug combination were also

subjected to statistical comparison (n = 4).
G Comparative analysis of the efficacy of drugs in primary PIT1-lineage samples following a 48-hour treatment at a concentration of 10 μM (n = 6).
H Dose–response assay of MDM2 inhibitor Idasanutlin in primary PIT1-lineage samples (n = 3). Data are shown as mean ± SEM. *$P < 0.05$, **$P < 0.01$, ***$P < 0.001$.

Table S1. The siRNA and primer sequences used in the research

Gene symbol	Application	Sequence
RBM39(R)	siRNA	GCCUCUAGCAAUUGGAUUATT
HNRNPA1(R)	siRNA	GUCGAAGUGGUUCCGGAAATT
HNRNPK(R)	siRNA	GACGUGCACAACCUUAUGATT
DDX17(R)	siRNA	GUCUUUACCUUCUCUUUCUTT
SRSF5(R)	siRNA	GGAUGCACAUCGACCUAAATT
HNRNPH1(R)	siRNA	GGGACACAGAUAUGUUGAATT
SRSF1(R)	siRNA	GCGUGAAGCAGGUGAUGUATT
PRPF8(R)	siRNA	GCUCAGCGAUCAGGAUUAATT
TARDBP(R)	siRNA	GGUCAAGAAAGAUCUUAAATT
UPF1(R)	siRNA	GGCCUUAACAAGAAGAGAATT
RBM39(R)	Primer-Forward	GCCGCTACAGAAGTCCCTAC
	Primer-Reverse	TGATGCTATGAGGCAACCCAA
HNRNPA1(R)	Primer-Forward	GGGATTTGCGTTTGTCACCTT
	Primer-Reverse	TTCGACCTCTCTGGCTGGAT
HNRNPK(R)	Primer-Forward	ATGCCAGTGTTTCAGTCCCAG
	Primer-Reverse	CAGCATCAGATTCGAGCGG
DDX17(R)	Primer-Forward	TCAAGGATGGTGGTCGGAGA
	Primer-Forward	GCTTCCATAGCCACTGCCAT
SRSF5(R)	Primer-Forward	ATGAGTGGCTGTCGAGTGTT
	Primer-Reverse	AACAGCGTCATCTGCATCCC
HNRNPH1(R)	Primer-Forward	CTTCCAGGGGAGGAGTACGG
	Primer-Reverse	GGCCATAAGTTTTCGTGGTGG
SRSF1(R)	Primer-Forward	AAACTGCCTACATCCGGGTT
	Primer-Reverse	GGCTTCTGCTACGACTACGG
PRPF8(R)	Primer-Forward	AGACAAAGGCAACAACCCCA
	Primer-Forward	GGGTCATGTCGAACACCCAT
TARDBP(R)	Primer-Forward	ACGAGCCTTTGAGAAGCAGA
	Primer-Forward	TCCACAAAGAGACTGGGCAA
GAPDH(R)	Primer-Forward	TCTCTGCTCCTCCCTGTTCT
	Primer-Reverse	ATCCGTTCACACCGACCTTC
GH(R)	Primer-Forward	ACCTCAGCCTCAGGGAAG
	Primer-Reverse	GCCCAGCAGAGAACTGTG
PIT1(R)	Primer-Forward	TAAACTAGATTAAATCAGCAACCC
	Primer-Reverse	TAGGAGTAAAGGCAATGCTATGAA
MDM2-mRNA(R)	Primer-Forward	TCAGGCAGGAGAAAGCGATG
	Primer-Reverse	CCAGTTCTCACGAAGGGTCC
Probe-based MDM2 (R)	Primer-Forward	TCCGACCACCGTGCTTCT
	Primer-Reverse	GAAACTTGGGACTCCGAACACA
Full-length Probe (R)	FAM	CCTCCAGGTTAGACCAAAACCATT
ΔExon3 Probe (R)	VIC	CCTCCAGATTATATTTTATATTGGACAG
Probe-based MDM2 (H)	Primer-Forward	TACTGATGGTGCTGTAAC
	Primer-Reverse	GATTCCTGCTGATTGACTAC

Full-length Probe (H)	FAM	TGCACAAAAAGACACTTATACTA
ΔExon3 Probe (H)	VIC	CAAGAGACCCTGGAAAATATATACC
MDM2-Exon2/Intron2(R)	Primer-Forward	GCAGGCGAGCGGAGAC
	Primer-Reverse	TGCATTTACGAAAAGAGACAACA
MDM2-Exon1/Exon3(R)	Primer-Forward	CTTCGCTCGACCTCCCGA
	Primer-Reverse	TCTCTTGTTCCGACGCTGG
MDM2-Exon1/Exon2(R)	Primer-Forward	CCTCCCGAGCGAAATGGTC
	Primer-Reverse	CAGAAGCACGGTGGTCGG
MDM2-Intron1(R)	Primer-Forward	GTAATGGAGGGAGGGGAGAGG
	Primer-Reverse	CTAACTTGACCAGCCCCACAG
MDM2-Exon2/Exon3(R)	Primer-Forward	TCTCCGACCACCGTGCTT
	Primer-Reverse	GTCTCTTGTTCCGACGCTGG
MDM2-Intron3(R)	Primer-Forward	CAGACTTAGGTGGCTGTAGCA
	Primer-Reverse	TAGCAAGCTGTTACCCACGAA
MDM2-Exon4/Exon5(R)	Primer-Forward	AAGCAGCAGCACATTGTGTA
	Primer-Reverse	GTTGACTTACAACCACTAAGTTTCT
MDM2-Intron4(R)	Primer-Forward	GAGCCTTCTGTGAGGTTGGTT
	Primer-Reverse	CACAGACAGAGGTGACAGACC
PARP-Exon8/Exon9(R)	Primer-Forward	GAAAGTGAACCGAGAAGGGGA
	Primer-Reverse	AGATACCCTTGCCAACACGA
PARP-Exon10/Exon11(R)	Primer-Forward	GATCCCAGCTTGAAGAGTCCA
	Primer-Reverse	TTGGGAGGCAGACAAAGTGAT
BAX(R)	Primer-Forward	ACCAAGAAGCTGAGCGAGTG
	Primer-Reverse	TCCACATCAGCAATCATCCTCT
P21(R)	Primer-Forward	CCCGAGAACGGTGGAACTTT
	Primer-Reverse	GAACACGCTCCCAGACGTAG
TP53(R)	Primer-Forward	TGAGGTTCGTGTTTGTGCCT
	Primer-Reverse	TCCGGGCAATGCTCTTCTTT
SD	Primer-Forward	TCTGAGTCACCTGGACAACC
SA	Primer-Reverse	ATCTCAGTGGTATTTGTGAGC
MDM2-Exon1/Exon3(H)	Primer-Forward	AAGGAAACTGGGGAGTCTTGAG
	Primer-Reverse	TGCACCAACAGACTTTAATAACTT
MDM2-Exon2/Exon5(H)	Primer-Forward	AACCACCTCACAGATTCCAGC
	Primer-Reverse	CTGCTGATTGACTACTACCAAGTTC
MDM2 mRNA(H)	Primer-Forward	GCCCTTCGTGAGAATTGGCT
	Primer-Reverse	AAGCCCTCTTCAGCTTGTGTT
GAPDH(H)	Primer-Forward	GCACCGTCAAGGCTGAGAAC
	Primer-Reverse	TGGTGAAGACGCCAGTGGA
ASO1(R)	ASO	CATAGATGCCTATATGTAA
ASO2(R)	ASO	TGCAGTTTTGTCAGTAGT
ASO3(R)	ASO	TAACCAACCTCACAGAAG
ASO1(H)	ASO	ACAGACATGTTGGTATTGCA
ASO2(H)	ASO	CCTCTTTCATAGTATAAGTG
ASO3(H)	ASO	GATGTACCTGAGTCCGATGA

Table S2. Clinical Information of the Samples

Patients ID[a]	Laboratory test of serum hormones[b]	Immunohistochemistry of hormones[c]	Lineage
Patient 1	Normal range	Negative	Null cell
Patient 2	PRL (+)	ACTH (+)	T-PIT
Patient 3	GH (+)	GH (+)	PIT1
Patient 4	GH (+), PRL (+)	GH (+)	PIT1
Patient 5	Normal range	ACTH (+)	T-PIT
Patient 6	Normal range	Negative	Null cell
Patient 7	Normal range	Negative	Null cell
Patient 8	Normal range	Negative	Null cell
Patient 9	Normal range	Negative	Null cell
Patient 10	Normal range	Negative	Null cell
Patient 11	Normal range	ACTH (+)	T-PIT
Patient 12	Normal range	Negative	Null cell
Patient 13	Normal range	Negative	Null cell
Patient 14	PRL (+)	PRL (+)	PIT1
Patient 15	Normal range	Negative	Null cell
Patient 16	PRL (+)	PRL (+)	PIT1
Patient 17	Normal range	FSH (+)	SF1
Patient 18	Normal range	FSH (+)	SF1
Patient 19	Normal range	FSH (+)	SF1
Patient 20	PRL (+)	Negative	Null cell
Patient 21	Normal range	FSH (+)	SF1
Patient 22	Normal range	FSH (+)	SF1
Patient 23	Normal range	Negative	Null cell
Patient 24	Normal range	GH (+)	PIT1
Patient 25	Normal range	FSH (+)	SF1
Patient 26	Normal range	Negative	Null cell
Patient 27	Normal range	Negative	Null cell
Patient 28	PRL (+)	Negative	Null cell
Patient 29	Normal range	Negative	Null cell
Patient 30	PRL (+)	Negative	Null cell
Patient 31	Normal range	FSH (+)	SF1
Patient 32	Normal range	Negative	Null cell
Patient 33	Normal range	Negative	Null cell
Patient 34	Normal range	Negative	Null cell
Patient 35	Normal range	Negative	Null cell
Patient 36	Normal range	Negative	Null cell
Patient 37	Normal range	Negative	Null cell
Patient 38	GH (+), PRL (+)	GH (+)	PIT1
Patient 39	GH (+)	GH (+)	PIT1
Patient 40	Normal range	Negative	Null cell
Patient 41	Normal range	FSH (+)	SF1

Patient 42	GH (+)	GH (+)	PIT1
Patient 43	GH (+)	GH (+)	PIT1
Patient 44	Normal range	Negative	Null cell
Patient 45	Normal range	Negative	Null cell
Patient 46	Normal range	Negative	Null cell
Patient 47	Normal range	GH (+)	PIT1
Patient 48	Normal range	FSH (+)	SF1
Patient 49	Normal range	FSH (+)	SF1
Patient 50	Normal range	FSH (+)	SF1
Patient 51	Normal range	Negative	Null cell
Patient 52	GH (+)	GH (+)	PIT1
Patient 53	Normal range	FSH (+)	SF1
Patient 54	Normal range	GH (+)	PIT1
Patient 55	Normal range	Negative	Null cell
Patient 56	Normal range	ACTH (+)	T-PIT
Patient 57	Normal range	PRL (+)	PIT1
Patient 58	Normal range	FSH (+)	SF1
Patient 59	Normal range	Negative	Null cell
Patient 60	Normal range	Negative	Null cell
Patient 61	Normal range	PRL (+)	PIT1
Patient 62	Normal range	ACTH (+)	T-PIT
Patient 63	Normal range	Negative	Null cell
Patient 64	Normal range	Negative	Null cell
Patient 65	Normal range	Negative	Null cell
Patient 66	Normal range	Negative	Null cell
Patient 67	Normal range	Negative	Null cell
Patient 68	PRL (+)	PRL (+)	PIT1
Patient 69	Normal range	GH (+)	PIT1
Patient 70	Normal range	FSH (+)	SF1
Patient 71	Normal range	Negative	Null cell
Patient 72	Normal range	GH (+)	PIT1
Patient 73	Normal range	Negative	Null cell
Patient 74	Normal range	Negative	Null cell
Patient 75	PRL (+)	PRL (+)	PIT1
Patient 76	Normal range	FSH (+)	SF1
Patient 77	Normal range	Negative	Null cell
Patient 78	Normal range	PRL (+)	PIT1
Patient 79	Normal range	Negative	Null cell
Patient 80	Normal range	Negative	Null cell
Patient 81	PRL (+)	Negative	Null cell
Patient 82	Normal range	Negative	Null cell
Patient 83	GH (+)	GH (+)	PIT1
Patient 84	Normal range	TSH (+)	PIT1
Patient 85	GH (+)	GH (+)	PIT1

Patient 86	Normal range	Negative	Null cell
Patient 87	PRL (+)	Negative	Null cell
Patient 88	Normal range	Negative	Null cell
Patient 89	Normal range	Negative	Null cell
Patient 90	GH (+), PRL (+)	GH (+)	PIT1
Patient 91	GH (+)	GH (+)	PIT1
Patient 92	Normal range	PRL (+)	PIT1
Patient 93	Normal range	Negative	Null cell
Patient 94	Normal range	Negative	Null cell
Patient 95	GH (+)	GH (+)	PIT1
Patient 96	Normal range	Negative	Null cell
Patient 97	Normal range	Negative	Null cell
Patient 98	Normal range	FSH (+)	SF1
Patient 99	Normal range	Negative	Null cell
Patient 100	Normal range	Negative	Null cell
Patient 101	Normal range	FSH (+)	SF1
Patient 102	Normal range	FSH (+)	SF1
Patient 103	Normal range	Negative	Null cell
Patient 104	PRL (+)	FSH (+)	SF1
Patient 105	Normal range	Negative	Null cell
Patient 106	Normal range	Negative	Null cell
Patient 107	GH (+)	GH (+)	PIT1
Patient 108	Normal range	Negative	Null cell
Patient 109	Normal range	Negative	Null cell
Patient 110	Normal range	TSH (+)	PIT1
Patient 111	Normal range	PRL (+)	PIT1
Patient 112	Normal range	Negative	Null cell
Patient 113	PRL (+)	GH (+)	PIT1
Patient 114	Normal range	Negative	Null cell
Patient 115	Normal range	Negative	Null cell
Normal PG 1	\	\	Normal
Normal PG 2	\	\	Normal
Normal PG 3	\	\	Normal
Normal PG 4	\	\	Normal
Normal PG 5	\	\	Normal
Normal PG 6	\	\	Normal
Normal PG 7	\	\	Normal
Normal PG 8	\	\	Normal
Normal PG 9	\	\	Normal
Normal PG 10	\	\	Normal

a: Normal PG (Normal pituitary gland tissue) was obtained from voluntary body donation without PitNETs;

b: The upper reference limit used to define abnormal pituitary hormone levels was: GH, 10 ng/mL; PRL, 30 ng/mL; ACTH, 50 pg/mL; TSH, 4.0 mIU/L; FSH, male 12.4 mIU/mL, female 20

mIU/mL; LH, male 8.6 IU/L, female 50 mIU/mL. The stalk effect can also lead to elevated PRL levels.

c: Positive expression was determined based on the pathological staining report.

Table S3. Clinical and phenotypical characteristics of 30 PIT1-lineage PitNETs

FUS expression	HIGH	LOW	Total	*p* Value
**Number of patients**	15	15	30	
**Age**	44.27 ± 11.42	38.27 ± 9.558		0.13
**Gender**				0.43
Male	6	4	10	
Female	9	11	20	
**PFS (in months)**	45.07 ± 32.58	36.27 ± 27.74		0.43
**Tumor size**				
Diameter MAX	28.45 ± 9.81	22.47 ± 9.74		0.10
Volume (cm^3, mean ± SD)	9.77 ± 9.42	4.49 ± 4.66		0.06
**Knosp grade**				0.87
0	2	1	3	
1	2	4	6	
2	2	2	4	
3	6	6	12	
4	3	2	5	
**Functional/Nonfunctional**				0.54
Functional	14	13	27	
Nonfunctional	1	2	3	
**Pathological classification**				0.30
**(Functional cases)**				
GH	9 (8)	9 (9)	18 (17)	
PRL	6 (6)	4 (3)	10 (9)	
TSH	0 (0)	2 (1)	2 (1)	
**Postoperative status**				0.03
Hypopituitarism	11	4	15	
Normal pituitary function	4	11	15	

Table S4. Clinical and phenotypical characteristics of 115 PitNETs

FUS expression	HIGH	LOW	Total	*p* Value
**Number of patients**	58	57	115	
**Age**	49.36 ± 10.25	44.00 ± 11.88		< 0.05
**Gender**				< 0.001
Male	41	15	56	
Female	17	42	59	
**PFS (in months)**	56.45 ± 29.03	49.35 ± 30.96		0.23
**Tumor size**				
Diameter MAX	33.69 ± 10.31	26.27 ± 9.79		< 0.001
Volume (cm^3, mean ± SD)	16.00 ± 16.85	7.56 ± 8.30		< 0.001
**Knosp grade**				0.34
0	0	4	4	
1	13	12	25	
2	12	9	21	
3	22	22	44	
4	11	10	21	
**Functional/Nonfunctional**				< 0.05
Functional	22	33	55	
Nonfunctional	36	24	60	
**Suprasellar invasion**				< 0.05
Invasive	50	38	88	
Non-invasive	8	19	27	
**Infrasellar invasion**				0.12
Invasive	21	13	34	
Non-invasive	37	44	81	
**Cystic degeneration**				0.17
Cystic degeneration	22	13	35	
Non-cystic degeneration	38	44	83	
**Visual impairment**				< 0.001
Visual impairment	44	24	68	
Unimpaired Vision	8	25	33	
**Intraoperative bleeding**	428.0 ± 606.1	141.3 ± 105.3		< 0.01
**Lost to follow up**	5	9	14	
**Postoperative status**				< 0.05
Hypopituitarism	36	22	58	
Normal pituitary function	17	26	43	

Table S5. Pathological characteristics of primary PitNETs samples

Sample ID	IHC staining for hormone granules	IHC staining for TF	Lineage
PIT1 Sample 1	GH (+), PRL (+)	PIT1 (+)	PIT1
PIT1 Sample 2	PRL (+)	PIT1 (+)	PIT1
PIT1 Sample 3	GH (+), PRL (+)	PIT1 (+)	PIT1
PIT1 Sample 4	GH (+), PRL (+)	PIT1 (+)	PIT1
PIT1 Sample 5	GH (+), PRL (+)	PIT1 (+)	PIT1
PIT1 Sample 6	GH (+), PRL (+)	PIT1 (+)	PIT1
PIT1 Sample 7	GH (+), PRL (+)	PIT1 (+)	PIT1
PIT1 Sample 8	GH (+), PRL (+)	PIT1 (+)	PIT1
SF1 Sample 1	LH (+), FSH (+)	SF1 (+)	SF1
SF1 Sample 2	LH (+), FSH (+)	SF1 (+)	SF1
SF1 Sample 3	LH (+), FSH (+)	SF1 (+)	SF1
SF1 Sample 4	LH (+), FSH (+)	SF1 (+)	SF1
SF1 Sample 5	LH (+), FSH (+)	SF1 (+)	SF1
SF1 Sample 6	LH (+), FSH (+)	SF1 (+)	SF1
SF1 Sample 7	LH (+), FSH (+)	SF1 (+)	SF1
SF1 Sample 8	LH (+), FSH (+)	SF1 (+)	SF1
T-PIT Sample 1	ACTH (+)	T-PIT (+)	T-PIT
T-PIT Sample 2	ACTH (+)	T-PIT (+)	T-PIT
T-PIT Sample 3	ACTH (+)	T-PIT (+)	T-PIT
T-PIT Sample 4	ACTH (+)	T-PIT (+)	T-PIT
T-PIT Sample 5	ACTH (+)	T-PIT (+)	T-PIT
T-PIT Sample 6	ACTH (+)	T-PIT (+)	T-PIT
T-PIT Sample 7	ACTH (+)	T-PIT (+)	T-PIT
T-PIT Sample 8	ACTH (+)	T-PIT (+)	T-PIT

Table S6. TOP 10 FUS-regulated downstream RNA targets

Gene Symbol	Log2 (fold change) of RIP[a]	Type of splicing[b]	*P* value	Description
Usp37	2.62	Skipping Exon	-0.22	Thiol-dependent ubiquitinyl hydrolase activity
Dzip3	2.57	Skipping Exon	-0.41	RNA binding, ubiquitin protein ligase activity
Hnrnph3	2.38	Skipping Exon	0.47	Nucleic acid binding, RNA binding
Pbrm1	2.26	Skipping Exon	-0.17	Chromatin remodeling, negative regulation of cell proliferation
Apc	2.17	Skipping Exon	-0.14	Wnt signaling pathway, Hippo signaling pathway
Mdm4	1.95	Skipping Exon	-0.21	p53 signaling pathway, microRNAs in cancer
Dnm1l	1.45	Skipping Exon	-0.13	Necroptosis, NOD-like receptor signaling pathway
Rbm5	1.39	Skipping Exon	-0.11	Metal ion binding, nucleic acid binding
Mdm2	1.21	Skipping Exon	-0.21	p53 signaling pathway, Ubiquitin mediated proteolysis
Mff	1.18	Skipping Exon	-0.19	Integral component of membrane

a: Log2 transformed expression in RIP-FUS relative to IgG control ($p < 0.05$).

b: Differential splicing analysis with rMATS using transcriptome data from the shFUS versus shNC groups ($p < 0.05$).

Table S7. Clinical-pathological characteristics of primary PitNETs cells

Sample ID	Functional /Nonfunctional	IHC staining for hormone granules	IHC staining for TF	Lineage
Sample 1	Nonfunctional	GH (+), PRL (+)	PIT1 (+)	PIT1
Sample 2	Functional	PRL (+)	PIT1 (+)	PIT1
Sample 3	Nonfunctional	GH (+), PRL (+)	PIT1 (+)	PIT1
Sample 4	Nonfunctional	GH (+), PRL (+)	PIT1 (+)	PIT1
Sample 5	Functional	PRL (+)	PIT1 (+)	PIT1
Sample 6	Functional	GH (+), PRL (+)	PIT1 (+)	PIT1
